# Overview and Future Perspectives on Tumor-Targeted Positron Emission Tomography and Fluorescence Imaging of Pancreatic Cancer in the Era of Neoadjuvant Therapy

**DOI:** 10.3390/cancers13236088

**Published:** 2021-12-02

**Authors:** Martijn A. van Dam, Floris A. Vuijk, Judith A. Stibbe, Ruben D. Houvast, Saskia A. C. Luelmo, Stijn Crobach, Shirin Shahbazi Feshtali, Lioe-Fee de Geus-Oei, Bert A. Bonsing, Cornelis F. M. Sier, Peter J. K. Kuppen, Rutger-Jan Swijnenburg, Albert D. Windhorst, Jacobus Burggraaf, Alexander L. Vahrmeijer, J. Sven D. Mieog

**Affiliations:** 1 Department of Surgery, Leiden University Medical Center, 2333 ZA Leiden, The Netherlands; f.a.vuijk@lumc.nl (F.A.V.); J.A.stibbe@lumc.nl (J.A.S.); R.D.Houvast@lumc.nl (R.D.H.); b.a.bonsing@lumc.nl (B.A.B.); C.F.M.Sier@lumc.nl (C.F.M.S.); P.J.K.Kuppen@lumc.nl (P.J.K.K.); kb@chdr.nl (J.B.); a.l.vahrmeijer@lumc.nl (A.L.V.); J.S.D.Mieog@lumc.nl (J.S.D.M.); 2 Department of Medical Oncology, Leiden University Medical Center, 2333 ZA Leiden, The Netherlands; s.a.c.luelmo@lumc.nl; 3 Department of Pathology, Leiden University Medical Center, 2333 ZA Leiden, The Netherlands; a.s.l.p.crobach@lumc.nl; 4 Department of Radiology, Leiden University Medical Center, 2333 ZA Leiden, The Netherlands; S.Feshtali@lumc.nl; 5 Department of Radiology, Section of Nuclear Medicine, University Medical Center Leiden, 2333 ZA Leiden, The Netherlands; l.f.de_geus-oei@lumc.nl; 6 Biomedical Photonic Imaging Group, University of Twente, 7522 NB Enschede, The Netherlands; 7 Percuros B.V., 2333 CL Leiden, The Netherlands; 8 Department of Surgery, Amsterdam UMC, Location AMC, 1105 AZ Amsterdam, The Netherlands; r.j.swijnenburg@amsterdamumc.nl; 9 Department of Radiology, Section of Nuclear Medicine, Amsterdam UMC, Location VUmc, 1081 HV Amsterdam, The Netherlands; ad.windhorst@amsterdamumc.nl; 10 Centre for Human Drug Research, 2333 CL Leiden, The Netherlands

**Keywords:** pancreatic ductal adenocarcinoma, targeted molecular imaging, positron emission tomography, near-infrared fluorescence imaging, neoadjuvant therapy, response monitoring, fluorescence guided surgery

## Abstract

**Simple Summary:**

Patients diagnosed with pancreatic cancer have a poor prognosis at time of diagnosis, with a 5-year survival rate of merely 10%. The only treatment with curative intent is surgical resection of the tumor and adjacent tumor-containing lymph nodes. To improve surgical outcome and survival, additional (imaging) tools are needed that support complete surgical tumor resection. Firstly, more accurate monitoring of tumor response to neoadjuvant treatment and subsequent determination of resectability is needed. Secondly, an imaging tool is needed for intraoperative guidance allowing accurate identification, delineation, and complete resection of the tumor and suspected lymph nodes. Therefore, both tumor-targeted PET/CT before surgery and real time fluorescence-guidance during surgery could be helpful to improve patient outcome. This review focusses on literature considering tumor-targeted PET/CT and near-infrared fluorescence (NIRF) imaging. Several tumor-targeted agents are under clinical evaluation, and several other promising agents are currently tested preclinically, both with promising results. Their additional diagnostic value and feasibility for future implementation in standard clinical care of PDAC has yet to be established in phase III clinical trials.

**Abstract:**

Background: Despite recent advances in the multimodal treatment of pancreatic ductal adenocarcinoma (PDAC), overall survival remains poor with a 5-year cumulative survival of approximately 10%. Neoadjuvant (chemo- and/or radio-) therapy is increasingly incorporated in treatment strategies for patients with (borderline) resectable and locally advanced disease. Neoadjuvant therapy aims to improve radical resection rates by reducing tumor mass and (partial) encasement of important vascular structures, as well as eradicating occult micrometastases. Results from recent multicenter clinical trials evaluating this approach demonstrate prolonged survival and increased complete surgical resection rates (R0). Currently, tumor response to neoadjuvant therapy is monitored using computed tomography (CT) following the RECIST 1.1 criteria. Accurate assessment of neoadjuvant treatment response and tumor resectability is considered a major challenge, as current conventional imaging modalities provide limited accuracy and specificity for discrimination between necrosis, fibrosis, and remaining vital tumor tissue. As a consequence, resections with tumor-positive margins and subsequent early locoregional tumor recurrences are observed in a substantial number of patients following surgical resection with curative intent. Of these patients, up to 80% are diagnosed with recurrent disease after a median disease-free interval of merely 8 months. These numbers underline the urgent need to improve imaging modalities for more accurate assessment of therapy response and subsequent re-staging of disease, thereby aiming to optimize individual patient’s treatment strategy. In cases of curative intent resection, additional intra-operative real-time guidance could aid surgeons during complex procedures and potentially reduce the rate of incomplete resections and early (locoregional) tumor recurrences. In recent years intraoperative imaging in cancer has made a shift towards tumor-specific molecular targeting. Several important molecular targets have been identified that show overexpression in PDAC, for example: CA19.9, CEA, EGFR, VEGFR/VEGF-A, uPA/uPAR, and various integrins. Tumor-targeted PET/CT combined with intraoperative fluorescence imaging, could provide valuable information for tumor detection and staging, therapy response evaluation with re-staging of disease and intraoperative guidance during surgical resection of PDAC. Methods: A literature search in the PubMed database and (inter)national trial registers was conducted, focusing on studies published over the last 15 years. Data and information of eligible articles regarding PET/CT as well as fluorescence imaging in PDAC were reviewed. Areas covered: This review covers the current strategies, obstacles, challenges, and developments in targeted tumor imaging, focusing on the feasibility and value of PET/CT and fluorescence imaging for integration in the work-up and treatment of PDAC. An overview is given of identified targets and their characteristics, as well as the available literature of conducted and ongoing clinical and preclinical trials evaluating PDAC-targeted nuclear and fluorescent tracers.

## 1. Introduction

Pancreatic cancer is one of the most lethal cancer types and is the third leading cause of cancer-related death in Europe, which is expected to rise even further within the next decades [1,2]. Pancreatic ductal adenocarcinoma (PDAC) is the most common subtype of pancreatic cancer and accounts for >90% of all pancreatic neoplasms [3]. Despite advances in surgical and systemic treatment, the 5-year overall survival (OS) rate remains approximately 10% [4]. This low survival rate is mostly caused by late detection of disease due to the late onset of symptoms [5]. Therefore, most patients are diagnosed with advanced stage disease, and only a minority (15–20%) of patients are eligible for treatment with curative intent [4,6,7].

The diagnostic workup for PDAC typically consists of a combination of CT for staging and endoscopic ultrasound with fine needle aspiration (EUS-FNA) or biopsy or endoscopic retrograde cholangiopancreatography (ERCP) to obtain histological confirmation of disease [8,9]. Recently, magnetic resonance imaging (MRI) has gained ground for the primary evaluation of local disease stage and vascular encasement by tumor tissue, as well as the characterization of distant metastases, especially in the peritoneal cavity and liver [10]. The role of ^18^F-FDG positron emission tomography (PET) combined with computed tomography (PET/CT) in the workup of pancreatic cancer remains controversial. The National Comprehensive Cancer Network (NCCN) consensus guideline states that FDG-PET/CT may be used per institutional preference; although, it is not a substitute for high-quality contrast-enhanced CT (ce-CT) [8]. The European Society for Medical Oncology (ESMO) states likewise and says the role of PET/CT should be further clarified [9]. The individual treatment plan is based on various clinical and radiological parameters, including tumor stage, the presence of metastatic disease, the extent of tumor invasion into major blood vessels, and the patient’s physical condition.

Determination of resectability of a pancreatic tumor with clear surgical margins is crucial, as only complete surgical resection of the tumor can provide curative-intent treatment. Constantly developing surgical techniques (e.g., robot-assisted surgery) and the clinical introduction of (neo)adjuvant therapy have significantly improved patient outcomes in the past decade, resulting in a 30–40% five-year OS after complete (R0) tumor resection, compared to 17.4% in 2011 [4,11,12]. The incomplete surgical resection rates (R1 or up) vary enormously in the available literature, between 20 and 70% of all pancreatic resections for malignant disease show positive surgical margins, which dramatically increase the rate of local and early recurrence of pancreatic cancer [6,13,14,15,16]. Aiming to increase the number of patients eligible for curative-intent resection and to further optimize surgical outcome, the combination of neoadjuvant induction therapy and adjuvant treatment has been under clinical investigation in the past years [4,17,18,19,20]. There are currently two combinations recommended as first-line (neo)adjuvant treatment regimens by the NCCN and ESMO: (modified) FOLFIRINOX (Folic acid, 5-Fluoruracil, Irinotecan, and Oxaliplatin) or gemcitabine plus nab-paclitaxel, the last is often combined with radiation therapy [8,9,21]. Since individual patient health status and morbidity highly influence the ability to receive (neo)adjuvant treatment, most well-considered multidisciplinary recommendations for duration and intensity of treatment are made within these standardized regimens or ongoing clinical trials for individual patients [4].

Focusing on neoadjuvant therapy (NT), the most clinical benefit could be gained within the borderline resectable and locally advanced patients; however, a standardized role in primary resectable disease should also be considered [22]. NT aims to slow disease progression, decrease tumor volume and local extensiveness, as well as eradication of potentially ‘occult’ micrometastases. NT, on one side, provides an extended time-window to detect rapid progressive disease, thereby potentially avoiding futile surgeries. On the other side, it provides a way to increase the eligibility for curative-intent resection, raise the percentage of radical resections (R0) and improve the surgical outcome [4,23,24]. The advantages of NT are underlined by the results of the recently published PREOPANC-1 trial. This trial compared clinical outcome and survival data of postoperative patients with resectable and borderline resectable disease who had received neoadjuvant or adjuvant therapy. Results showed improved survival and higher complete surgical resection (R0) rates in the neoadjuvant therapy arm, with a 30% increase in R0 resections (71% vs. 40%) and a 2-month prolonged median survival (16 vs. 14 months) [18]. More recently, the recruitment of patients for its successor, the PREOPANC-II trial (NTR7292) was completed. In this trial neoadjuvant treatment with FOLFIRINOX was compared to neoadjuvant Gemcitabine-Radiotherapy followed by adjuvant Gemcitabine in patients with (borderline) resectable disease [25].

To date, accurate assessment of response to (neo)adjuvant treatment remains challenging, which is a crucial step in re-staging and determination of resectability [26,27]. Currently, treatment response is monitored with CT-imaging, which is evaluated by radiologists using the internationally standardized RECIST 1.1 criteria [28,29]. These criteria focus on a percentual change in tumor dimensions (longest diameter), which are used to determine therapy response: a complete response (CR), partial response (PR), progressive disease (PD), or stable disease (SD) [28]. Although the role of this approach for assessment of response is limited, besides overestimation of tumor size on CT, the change in tumor attenuation is of limited value in the prediction of resectability, due to the inability to differentiate treatment-related necrosis, therapy-induced fibrosis (TIF), and tumor-associated pancreatitis (TAP) from residual vital tumor tissue in the pancreas [26,30,31]. Cassinotto et al. concluded ce-CT lacks the sensitivity and performance for accurately monitoring treatment response, showing that the diagnostic performance of ce-CT to predict resectability decreased after neoadjuvant treatment (58% vs. 83%) [32]. Ferrone et al. showed similar results, stating that ce-CT after FOLFIRINOX treatment no longer adequately predicts resectability of the tumor [33]. These results underline the need for improved imaging methods for assessment of therapy response, since this is pivotal for accurate (re)staging and determination of tumor resectability [31,34,35]. In addition to conventional CT-imaging, molecular-based FDG-PET/CT-imaging has been evaluated for monitoring of (neo)adjuvant treatment response in various malignancies, including PDAC [36,37,38]. Despite some favorable results, the main disadvantage of FDG-based PET/CT-imaging of pancreatic tissue is the increased uptake seen in TAP, complicating adequate differentiation between the remaining tumor and adjacent benign tissue [39]. Molecular-targeted tumor imaging has the potential to overcome these challenges by selectively targeting tumor biomarkers overexpressed on or in close proximity to PDAC cells, resulting in high tumor-specific signals with minimal background accumulation in surrounding normal tissue.

Following induction treatment and restaging, the next vital steps for curative intent resection are: intraoperative visualization and delineation of the tumor to its anatomical demarcations and relations with vital structures, identification of suspect tumor-containing lymph nodes, as well as assessment of the surgical margins for residual vital tumor. However, the complex and heterogeneous tumor characteristics of PDAC with its extensive desmoplastic reaction and locoregional changes resulting from NT as well as its retroperitoneally anatomical location make this very challenging [31]. Near-infrared fluorescence (NIRF) imaging, also called fluorescence-guided surgery (FGS), a novel technique, can offer a solution by providing real-time intraoperative guidance by enhancing visual contrast for localization of the tumor and discrimination between malignant and benign tissue [40,41]. FGS uses a fluorescent dye conjugated to a molecular tracer designed to bind specific molecular features on (tumor)-target cells (i.e., tumor tissue, tumor stroma, etc.) [40]. Aiding a surgeon with a tool that enhances intraoperative surgical navigation to detect tumor, lymph node, and metastatic deposits in real-time, might eventually result in fewer incomplete surgical resections (R1) and improve surgical outcome and OS in the near future.

Multiple molecular targets, or *biomarkers*, expressed by PDAC, have been identified in previous studies. These biomarkers form the basis for tumor-targeted nuclear and fluorescence imaging in various malignancies, including PDAC [34,42,43,44]. Molecular imaging of oncological targets has been of particular interest in the past decade: multiple (pre)clinical trials have shown promising results for PDAC-targeted PET/CT and NIR-imaging, for diagnostic as well as therapeutic purposes [45,46,47,48].

**Figure 1:**
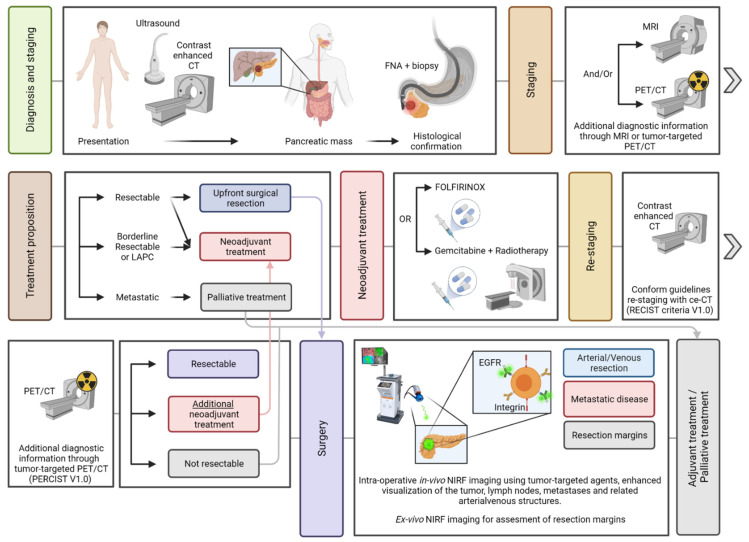
An overview is given of the patient journey from initial presentation to follow-up. Incorporation of molecular imaging in several stages of disease is presented. Abbreviations: CEA(CAM) = Carcinoembryonic antigen; EGFR = Epidermoid growth factor receptor; LAPC = Locally advanced pancreatic cancer; PDAC = Pancreatic ductal adenocarcinoma; RECIST = Response Evaluation Criteria in Solid Tumors; PERCIST = Positron Emission Tomography (PET) Response Criteria in Solid Tumors; NIRF = Near-Infrared Fluorescence Imaging. Created with BioRender.com.

This review covers the current strategies, obstacles, and developments in molecular-targeted tumor imaging, focusing on the potentials and possibilities of integrating nuclear and fluorescence imaging in the work-up of PDAC (Figure 1). Previous and future (pre)clinical trials with tumor specific imaging agents, have been selected and presented to address the potential value of nuclear and fluorescence imaging in PDAC in the future.

A comprehensive literature search in the PubMed database and (inter)national clinical trial registers (clinicaltrials.gov; clinicaltrialsregister.eu; trialregister.nl) was performed to collect all relevant preclinical and clinical papers regarding either nuclear (PET/CT) or fluorescence imaging of PDAC from the past 15 years. Data of eligible articles were reviewed and summarized in text and tables.

## 2. Molecular Targets in Pancreatic Ductal Adenocarcinoma (PDAC)

### 2.1. Target Characteristics for PDAC Imaging

Considering their pivotal roles in the onset and progression of cancer, the identification of tumor-specific molecular markers, located on tumor cells or within the tumor microenvironment (TME), for diagnostic or therapeutic targeting, has been a major focus in cancer research. A wide range of tumor-specific biomarkers for PDAC have been identified, which will be discussed in the following paragraphs, and are depicted in Table 1. The identified biomarkers are listed according to alphabetical coherence along with their molecular classification and associated function. An in-depth overview of the results of preclinical and clinical studies for each biomarker can be found in, respectively, Table A1 and Table A2.

The ‘ideal’ target for tumor-targeted imaging has several characteristics, as described by van Oosten et al. [49]. An ideal target for PDAC imaging should be located on the cell membrane or within the extracellular matrix (ECM) in proximity of the tumor. Another category is intracellularly located targets, which possess enzymatic biological functions and are enzymatically activated. Furthermore, they should have a diffuse distribution and strong expression on the tumor cells, stroma, or on precursor lesions, including pancreatic intraepithelial neoplasia’s (PanINs). In addition, an ideal target should have an absent or minimal expression on healthy pancreatic tissue as well as on tumor-associated pancreatitis (TAP), which is commonly present, proximate to, and hard to distinguish from PDAC [31,44]. Furthermore, enzymatic activity could be advantageous for application of locally activated imaging agents. Lastly, internalization of the target-tracer complex, which could facilitate intracellular tracer accumulation is a characteristic of interest as it could result in selective tumor cell uptake and enhanced signals.

### 2.2. Overview of PDAC-Associated Molecular Targets for Imaging Purposes

#### 2.2.1. CA19.9

Carbohydrate antigen 19.9 (CA19.9) is a glycan attached to membrane-bound proteins and released by pancreatic (cancer) cells. CA19.9 plays a vital role in cell-to-cell recognition processes and high levels are associated with tumor progression, invasion, and metastasis [50]. Serum CA19.9 levels, which are increased in >70% of PDAC patients, are clinically used as a biomarker for diagnosis and monitoring of PDAC. Moreover, serum CA19-9 levels are correlated with stage of disease and OS [51,52]. On a tissue level, CA19.9 is aberrantly expressed in 70–90% of PDAC, and to a lesser extent in precursor lesions [53,54,55]. Nevertheless, elevated serum levels of CA19.9 and (over)expression are both observed in healthy pancreatic tissue, (chronic) pancreatitis, although, in varying levels of expression, as well as other hepatobiliary diseases, such as biliary tree obstruction [53,54,55]. This may potentially hamper adequate tumor identification and delineation. In addition to CA19-9, several CA19-9-related glycans are highly expressed on PDAC tissues, while expression on chronic pancreatitis and healthy pancreatic parenchyma is low or absent. Due to their amplified expression on the outermost layer of the tumor cell, tumor-associated glycans may provide several advantages for tumor targeting beyond proteins [56].

#### 2.2.2. Cathepsin-E

Cathepsin-E (Cath-E) is an intracellular located aspartic proteolytic enzyme, belonging to a larger group of cathepsins, which recycle proteins for cellular homeostasis. It is assumed that Cath-E downregulates the body’s immune response and promotes protein turnover [57]. Its carcinogenic function as well as the mechanism resulting in overexpression of Cath-E in PDAC and other cancer types is largely unknown [57]. Cath-E is expressed in >90% of PDAC as well as in pancreatic precursor lesion, while its expression in healthy pancreatic tissue and pancreatitis is absent or low. [57,58,59]. Considering its high and diffuse expression in early pancreatic cancer lesions as well as PDAC, Cath-E is a potentially interesting target for early PDAC detection. A drawback of targeting cathepsins is its dependence on internalized, protease-activatable tracers, which are essential for adequate target visualization [58,60,61].

#### 2.2.3. CDCP-1

Cub-domain containing protein-1 (CDCP-1) is a transmembrane glycosylated receptor protein present on epithelial cells. CDCP-1 regulates cell-to-cell adhesion and interacts with carcinogenic pathways, which promote tumor invasiveness and metastasis. Overexpression of CDCP-1 in PDAC as well as various other cancers is correlated with poor prognosis [62]. CDCP-1 is overexpressed in >90% of PDAC [62]. Pancreatic precursor lesions express CDCP-1 low to moderately, making it a potentially interesting target for early PDAC detection. CDCP-1 has been a target of interest for targeted-imaging as well as therapy in various cancers [63,64].

#### 2.2.4. CEA/CEACAM-5

Carcinoembryonic antigen (CEA), or CEACAM-5 is a cell adhesion molecule anchored to the cell membrane and is involved in cellular ECM adhesion, motility, and inhibition of apoptosis [52]. Like CA19.9, CEA is clinically used as a serum biomarker for diagnosis and monitoring of PDAC. CEA serum levels are increased in 40–70% of all PDAC patients, while CEA is overexpressed on the cell membrane in 70–85% of PDAC cases. Of note, CEA is virtually not expressed on healthy pancreatic tissue and moderately expressed in pancreatitis, strengthening its potential as a PDAC-specific target [44,54,65]. Anti-CEA targeted antibodies have been evaluated predominantly in PDAC-targeted fluorescence imaging and to a lesser extent in PET/CT-imaging, demonstrating tumor identification in both preclinical and clinical settings [66,67,68,69,70,71].

#### 2.2.5. EGFR

Epidermal growth factor receptor (EGFR) is a transmembrane tyrosine-kinase receptor (TKR) for epidermal growth factor (EGF). EGFR is expressed on the cell membrane of various normal human tissues, while overexpression is observed in several types of cancer, including PDAC. EGFR plays a key role in the transition from normal epithelial to neoplastic epithelial cells and overexpression in cancerous tissues results in activation of pathways involved in cell proliferation, survival, invasion, and metastasis, as well as neoangiogenesis, which classifies EGFR overexpression as an indicator for advanced disease, poor OS, and presence of metastases [72,73]. Overexpression on the cell membrane of PDAC cells varies from 70–90%, while EGFR has been detected to a lesser extent in pancreatic precursor lesions and is absent in normal pancreatic parenchyma [42,73]. Overexpression of EGFR expression in PDAC as well as its precursor lesions has made it a viable target for tumor-targeted imaging and therapy. Although targeted therapy using anti-EGFR antibodies (cetuximab, and panitumumab) was moderately effective in PDAC, their employment as targeting vehicles for NIR-fluorescence imaging has allowed clear tumor delineation in both preclinical and clinical settings [42,44,72,74,75].

#### 2.2.6. Endoglin

Endoglin is a component of the transforming growth factor-beta receptor complex (TGF-β) and is mainly expressed on the membrane of vascular endothelial cells. Endoglin, as a TGF-β co-receptor, modulates the response to the signaling cascade upon binding of TGF-β, rather than initiating the signaling cascade like the TGF-β receptor itself [76]. This signaling cascade mediates tumor invasiveness as well as metastatic spread through induction of (neo)angiogenesis, cell migration, and proliferation. Therefore, endoglin overexpression is related to significantly poorer OS [77]. Endoglin is diffusely upregulated in vascular endothelial PDAC cells, whereas vascular endothelial cells in healthy pancreatic tissue do not express endoglin [77,78]. Endoglin expression of vascular endothelial cells in pancreatitis is unknown. The applicability of targeting endoglin for molecular imaging of PDAC has to be demonstrated, since exact expression profiles of endoglin on PDAC precursor lesions as well as pancreatitis is not specifically studied, which could hamper its diagnostic value.

#### 2.2.7. EpCAM

Epithelial cell adhesion molecule (EpCAM) is a transmembrane protein that mediates epithelial cell-to-cell adhesions. Its carcinogenic function is known to promote tumor growth, metastatic spread, and functions as an oncogenic signaling protein. EpCAM is expressed by the majority of healthy epithelial tissues while being overexpressed in a subset of human carcinomas, including PDAC. EpCAM overexpression is seen in roughly 56–78% of all PDAC and to a lesser extent in pancreatic precursor lesions [44,79,80]. Overexpression of EpCAM in pancreatitis and healthy pancreatic tissue is varying, and conflicting profiles are seen in the literature, deemed minimal by Fong et al., whereas de Geus et al. demonstrate moderate expression profiles in healthy pancreatic tissue. Although EpCAM-targeted imaging tracers have been successfully evaluated for breast cancer delineation [81], their applicability for PDAC remains to be elucidated.

#### 2.2.8. FAP

Fibroblast Activation Protein-α (FAP) is a transmembrane protein, functioning as a serine protease and plays an important role in reactive fibroblasts, promoting angiogenesis and altering the extracellular matrix (ECM), which are crucial for tumor progression. FAP expression is only expressed by cancer-associated fibroblasts (CAF) present in the stromal compartment and in roughly 75% of the PDAC lesions. Moreover, low expression is seen in resting fibroblasts present in healthy pancreatic tissue, while moderate FAP expression is observed in patients with signs of pancreatitis [82]. Because PDAC is characterized by its prominent and dense stroma that mainly consists of CAFs, FAP is a high potential target for high-contrast tumor-targeted imaging of PDAC. Up until now, mainly applications for primary staging (PET/CT) have been investigated, showing promising results in late-stage clinical trials [83,84]. It is currently, one of the most promising targets for PDAC imaging and therapy.

#### 2.2.9. Fibronectin

Fibronectin is a high-molecular weight protein of the ECM that interacts with fibrins, integrins, and collagens, through which it is involved in cell-to-cell adhesion. Fibronectin overexpression by cancer-associated fibroblasts (CAFs) has been correlated to promote angiogenesis, metastatic spread, and resistance to chemotherapy [85]. Overexpression is correlated to advanced stage of disease. Fibronectin is overexpressed in >80% of PDAC, whereas no overexpression in normal pancreatic parenchyma is seen [86,87]. Expression profiles in precursor lesions and pancreatitis have not been evaluated. Assessment hereof, could support further evaluation of fibronectin as a target for PDAC imaging.

#### 2.2.10. GRP78

Glucose-Regulated Protein-78 (GRP78) is a chaperone signaling protein normally located in the endoplasmic reticulum. In the case of neoplastic cells, it is translocated to the cell membrane, functioning as co-receptor for various proteins, including integrins. GRP78 has a dedicated function in engaging endogenous cytoprotective processes, thereby promoting tumor cell survival pathways [88]. Overexpression of GRP78 is correlated to increased tumor growth, therapeutic resistance, and metastases [89,90]. GRP78 expression is upregulated on the cell membrane of PDAC cells and to a lesser extent in precursor lesions, while it is located in the endoplasmic reticulum in normal pancreatic parenchyma, which makes it an interesting target for targeted PET/CT, since the translocation of this protein from the ER to the cell membrane is solely seen on malignant pancreatic cells. Targeting GRP78 has already been evaluated in a preclinical setting for the monitoring response of GRP78 targeted-NT using small animal PET, demonstrating its value for evaluating disease course and therapeutic efficacy at the earliest stages this treatment [91,92].

#### 2.2.11. Integrins

Integrins are considered cell adhesion molecules and are the predominant receptors of the ECM, located on the cell membrane, binding various ligands, for example, RGD-sequences, fibronectin, and laminin. Upon binding their ligands, they activate signal transduction pathways mediating cell-to-cell and cell-to-ECM adhesion as well as cell migration. Integrin overexpression could therefore promote tumor progression and metastatic spread. Overexpression of various integrins, including αvβ3, αvβ5, and αvβ6, is seen in PDAC and correlates with more aggressive disease and decreased survival [93]. From the aforementioned PDAC-associated integrins, expression and function of integrins αvβ3 and αvβ6 have been most extensively evaluated. Integrin αvβ3 is expressed on stromal and endothelial cells in ~60% of PDAC, whereas integrin αvβ6 is expressed on the epithelial cells of 80–90% of PDAC lesions [93,94,95,96,97]. Integrin αvβ3 shows low to moderate expression on normal pancreatic tissue and moderate expression in pancreatitis [97]. Whereas integrin αvβ6 shows low expression on normal pancreatic tissue and moderate expression in pancreatitis [42,44,96,98]. Integrins possess favorable characteristics for tumor-targeted imaging, since targeting could take place with small-molecular sized peptides, which are easily modifiable and are able to pass biological and physical barriers essential for effective target binding [99,100]. Avβ3/αvβ6 peptide sequences have been developed in different configurations, such as linear, cyclic, or cystine knotted, for optimization of specific target binding affinity and elimination pattern [101].

#### 2.2.12. Mesothelin

Mesothelin is a GPI-anchored protein, present in the vast majority of mesothelial cells, in which it functions as an adhesion molecule and mediates cell-to-cell adhesion. Overexpression promotes cell proliferation, migration, and metastasis, while simultaneously interfering with pathways initiating cell apoptosis. Mesothelin is strongly and diffusely overexpressed in >90% of PDAC, while it is minimally expressed in pancreatitis and normal pancreatic parenchyma [102,103]. Considering its expression profile, mesothelin possesses favorable characteristics for high-contrast targeted imaging of PDAC lesions, but to a lesser extent for early PDAC detection, given its minimal expression on precursor lesions [104,105].

#### 2.2.13. MT1-MMP/MMP-14

Membrane type 1 matrix metalloproteinase (MT1-MMP, and MMP-14) is a cell membrane-associated endopeptidase involved in degradation of the ECM of stromal cells and facilitates cell migration, tumor invasiveness, and resistance to chemotherapy. Collagen-mediated overexpression of MT1-MMP is observed in the extensive desmoplastic regions of PDAC [106,107]. MT1-MMP is overexpressed in 75% of PDAC and to a lesser extent in precursor lesions and pancreatitis, while being absent in normal pancreatic parenchyma [108]. Since MT1-MMP plays a key role in establishing the desmoplastic reaction in pancreatic cancer, and subsequent tumor progression, it is of particular interest for investigation as a target for tumor-targeted diagnostic and therapeutic applications [109].

#### 2.2.14. Mucin-1

Mucin-1 is a high-molecular weight, transmembrane glycoprotein, present on the epithelium of the pancreas, liver, breast, kidneys, lungs, and reproductive organs, on which it contributes to a protective mucus barrier. Mucin-1 overexpression in PDAC is associated with resistance to cytotoxic drugs, tumor invasiveness, metastasis, and increased cell proliferation. Mucin-1 is aberrantly expressed in its under-glycosylated form on the basolateral membrane in 90% of epithelial PDAC cells and, to a lesser extent, in precursor lesions. In normal pancreatic parenchyma, Mucin-1 is expressed in heavily glycosylated form on the apical surface of epithelial cells [110,111,112]. Targeting moieties that recognize the tumor-associated, under-glycosylated Mucin-1 form are of particular interest for high-contrast (early) diagnostic imaging and monitoring of PDAC [113].

#### 2.2.15. NTSR1

Neurotensin receptor-1 (NTSR1) is a G-protein coupled receptor for neurotensin located on the cell membrane of cells of the upper-GI tract. In PDAC, NTSR1 is associated with activation of carcinogenic pathways resulting in cellular survival and inhibition of apoptosis. Therefore, NTSR1 overexpression in PDAC has shown to be correlated with more advanced disease [114]. NTSR1 is highly expressed in PDAC (~79–88%), with a low expression in normal pancreatic parenchyma and pancreatitis [115]. Since NTSR1 expression is selectively upregulated in PDAC, it possesses favorable characteristics for PDAC delineation. Several (pre)clinical trials have been conducted evaluating the performance of NTSR1 targeted peptides for tumor-targeted PET/CT as well as NIR-fluorescence imaging [115,116,117,118].

#### 2.2.16. PSMA

Prostate-specific membrane antigen (PSMA) is a cell membrane-associated enzyme, functioning as carboxypeptidase, thereby degrading protein or peptide bonds. PSMA is believed to be involved in induction of tumor neoangiogenesis; however, the exact mechanism remains unclear [119]. PSMA is expressed in 68% of the tumor-associated neovasculature in PDAC, and to a lesser extent on tumor cells, while no PSMA overexpression in healthy pancreatic tissue and pancreatitis is reported [119,120,121]. PSMA-targeted imaging has already shown promising results for diagnostic and therapeutic application in prostate cancer [122,123]. Given its tumor-specific abundance in PDAC, PSMA may be a high-potential target for high-contrast tumor-targeted imaging of PDAC [124,125,126].

#### 2.2.17. TF

Tissue factor (TF) is a cytokine-receptor for factor VII and an initiator of the coagulation cascade through factor X. TF contributes to tumor growth, angiogenesis, metastatic spread, and thrombogenesis and is correlated with advanced disease stage, histological grade, and poor OS in PDAC [127,128,129]. TF overexpression in PDAC is observed in 50–90% of cases, while it is moderately expressed in pancreatic precursor lesions. Low/moderate expression is seen in pancreatitis, whereas normal pancreatic parenchyma does only minimally express TF [127]. Further evaluation with the available TF-targeted small molecule inhibitors is warranted to address the potential for early detection and monitoring of PDAC [130,131,132].

#### 2.2.18. TfR1

Transferrin receptor-1 (TfR1) is an ion-channel coupled receptor for transferrin (Tf) that plays a key role in the cellular iron homeostasis of normal cells in the body and modulates cell growth. Overexpression of TfR1 is seen in various malignancies, since increased proliferation requires cell growth and, consequently, enhanced iron homeostasis. Overexpression of TfR1 has been correlated with advanced tumor stage and poor prognosis [133,134]. TfR1 is overexpressed in >90% of PDAC tissue, whereas expression in healthy pancreatic tissue is minimal [135]. Since TfR1 has been highly overexpressed in PDAC, it could be a target of interest for therapeutic and molecular imaging purposes. Nevertheless, more detailed information of TfR1 expression profiles in precursor lesions is warranted before evaluating its potential as a target for diagnostic PDAC imaging [136,137].

#### 2.2.19. uPA/uPAR System

Urokinase-plasminogen activator receptor (uPAR) is a GPI-anchored receptor that localizes urokinase-plasminogen (uPA) to the cell. Activation of uPA promotes degradation of the ECM and initiates angiogenesis and metastatic spread. uPAR is correlated with more aggressive disease, poor prognosis, and decreased OS. uPAR is overexpressed on 67–80% of PDAC lesions, by neoplastic, endothelial, as well as stromal cells. Overexpression is to a lesser extent seen in precursor lesions, while expression in healthy pancreatic tissue is minimal [138,139,140]. Considering its expression profile on both epithelial and stromal cells, significant tumor uptake is observed, making the uPA/uPAR system a particularly interesting target for molecular imaging PDAC [42,44].

#### 2.2.20. VEGFR/VEGF-A

Vascular endothelial growth factor receptors (VEGFR1 and VEGFR2) are TKRs present on vascular endothelial healthy and cancer cells. Their ligand, vascular endothelial growth factor-A (VEGF-A), is the most well-known angiogenic growth factor and abundantly present on vascular endothelial cells, in which it promotes neoangiogenesis. VEGF-A binds to VEGFR-2, which is overexpressed on the cell membrane of most gastro-intestinal cancers (GI), including PDAC [141]. Although PDAC is known as a hypovascular cancer type, angiogenesis is an essential process for supplying sufficient levels of oxygen and nutrients [142]. VEGFR-2 is overexpressed in >70% of vascular endothelial cells in PDAC and moderately overexpressed in pancreatitis, whereas no overexpression of VEGFR-2 expression is seen normal pancreatic parenchyma [143]. As VEGFR-2 expression is limited to the vascular endothelium and expression on pancreatitis is moderate, VEGFR-2 has less favorable characteristics for targeted PET/CT-imaging for primary diagnosis or response monitoring [34].

### 2.3. The Effect of (Neo)Adjuvant Treatment on Target Expression

A possible ‘side-effect’ of neoadjuvant treatment is the alteration of target expression on the tumor cells (differential expression), which may have direct consequences for the diagnostic performance of targeted imaging. Therefore, evaluation of the extent of this differential expression is a pivotal step to evaluate a tumor target. In a small cohort study, Tummers et al. demonstrated a significantly changed expression profile of CEA but not of αvβ6 in patients treated with neoadjuvant chemoradiation [42]. Vuijk et al. analyzed the differential expression pattern of neoadjuvant FOLFIRINOX treated PDAC tissue specimens on integrin αvβ6, CEA, mesothelin, PSMA, uPAR, FAP, Integrin Subunit Alpha 5 (ITGA5), and EGFR [31]. Except for uPAR, FAP, ITGA5, and EGFR, which were excluded due to low expression profiles, all analyzed targets showed a significantly higher expression in PDAC compared to tumor associated pancreatitis (TAP) and normal surrounding pancreatic tissue, while therapy induced fibrosis (TIF) showed no expression of integrin αvβ6, CEACAM5, and mesothelin. Integrin αvβ6, CEACAM5, mesothelin, and PSMA have the potential to distinguish vital PDAC from surrounding fibrotic tissue after neoadjuvant FOLFIRINOX treatment, strengthening their high potential as targets for molecular imaging of PDAC in a clinical setting.

### 2.4. Summary

As shown in the abovementioned sections, twenty different PDAC (-associated) targets have been identified. Of these targets, the tumor cell targets: CEACAM, EGFR, integrin αvβ6, mesothelin, NTSR1, PSMA, TF, uPA/uPAR, and VEGFR/VEGF-A; and stromal targets: FAP, Fibronectin, MT1-MMP/MMP-14 and Integrin αvβ3 own most of the characteristics characterizing the ‘ideal’ imaging target, as compiled by Oosten et al. [49]. Furthermore, since PDAC is known for its extensive desmoplastic reaction, causing PDAC lesions to consist for >90% of stromal tissue [144], targets expressed by the ECM (FAP, Fibronectin, MT1-MMP/MMP-14 and Integrin αvβ3) are of particular interest compared to epithelial markers (CDCP-1, EpCAM and Integrin αvβ6). Of these mentioned, a minority has been evaluated in clinical trials, which can mostly be attributed to the extensive, costly, and time-consuming preclinical research required for construction of suitable targeted tracers, which is followed by the preclinical validation and feasibility testing in animal models to assess the potential for further clinical translation.

**Table 1:** Overview of (pre)clinical evaluated PDAC biomarkers for molecular imaging (PET/CT -NIR-fluorescence).

	Target	Biological Function (Subtype)	Biological Effect Related to Expression by Tumor-(Associated) Cells	Location, Expression on Pancreatic Cell-Type	Target Expression in PDAC (% of +)	Advantages for PDAC Imaging	Disadvantages for PDAC Imaging	Expression Profile (0/−/+/++)	Ref.
	**CA19.9**	Glycan	Cell-to-cell recognition processes	Cell membrane, (neoplastic) pancreatic cells	70–90%	Diffuse, high expression in PDAC Moderate expression in precursor lesions	High expression in pancreatitis Moderate expression in healthy pancreatic tissue	NPT: + Pancreatitis: ++ Precursor lesions: −/+ PDAC: ++	[53,54,55]
	**Cathepsin-E**	Hydrolytic aspartic protease	Regulation of immune response, protein turnover, induction of apoptosis	Intracellular, (neoplastic) pancreatic cells	~92%	Diffuse, high expression in PDAC Moderate expression in Precursor lesions No expression in healthy pancreatic tissue	Low expression in pancreatitis	NPT: 0 Pancreatitis: − Precursor lesions: +/++ PDAC: ++	[57,59]
	**CDCP-1**	Glycosylated receptor protein	Cell proliferation, tumor invasiveness, metastasis	Cell membrane, (neoplastic) pancreatic epithelial cells	~92%	Diffuse, varying expression in PDAC No expression in healthy pancreatic tissue Low/Moderate expression in precursor lesions	No data available of expression profile in pancreatitis	NPT: 0 Pancreatitis: N/A Precursor lesions: −/+ PDAC: −/+/++	[145,146]
	**CEA**	Cell Adhesion Molecule	Oncogenic signaling protein, inhibition of apoptosis	Cell membrane, (neoplastic) pancreatic cells	70–85%	Diffuse, high expression in PDAC Moderate expression in precursor lesions No expression in healthy pancreatic tissue	Moderate expression in pancreatitis	NPT: 0 Pancreatitis + Precursor lesions: + PDAC: ++	[44,65,147]
	**EGFR**	Tyrosine kinase Receptor (TKR)	Cell proliferation, metastasis, tumor angiogenesis	Cell membrane, (neoplastic) pancreatic cells	69–90%	Diffuse, high expression in PDAC> Moderate expression in precursor lesions Low expression in healthy pancreatic tissue	No data available of expression profile in pancreatitis	NPT: − Pancreatitis: N/A Precursor lesions: + PDAC: ++	[42,44]
	**Endoglin**	Co-receptor for TGF-β	Tumor angiogenesis, tumor growth, metastasis	Cell membrane, (neoplastic) pancreatic vascular endothelial cells	N/A	Diffuse, varying expression in PDAC, depending on tumor aggressiveness/stage Low expression in precursor lesions No expression in healthy pancreatic tissue	No data available of expression profile in pancreatitis	NPT: 0 Pancreatitis: N/A Precursor lesions: −/+ PDAC: −/+/++	[77,78]
	**EpCAM**	Cell Adhesion Molecule	Cell proliferation, metastasis, oncogenic signaling protein	Cell membrane, (neoplastic) pancreatic epithelial cells	56–78%	Diffuse, moderate expression in PDAC Low/Moderate expression in precursor lesions Low/Moderate expression in pancreatitis	Low/Moderate expression in healthy pancreatic tissue	NPT: −/+ Pancreatitis: −/+ Precursor lesions: −/+ PDAC: +/++	[44,80,148]
	**FAP-α**	Cell membrane associated enzyme	Fibroblast activation, promoting angiogenesis	Cell membrane, Cancer Associated Fibroblasts (CAFs) in stroma	73–76%	Diffuse, high expression by CAFs in PDAC Low expression in healthy pancreatic tissue	Moderate expression in pancreatitis No data available of expression profile in precursor lesions	NPT: − Pancreatitis + Precursor lesions: N/A PDAC: ++	[82]
	**Fibronectin (FN)**	Component of ECM	Cell proliferation, metastasis, resistance to chemotherapy	Cell membrane, pancreatic fibroblastic cells and CAFs	~85%	Diffuse, high expression by CAFs in PDAC No overexpression by fibroblasts within healthy pancreatic tissue	No data available of expression profile in pancreatitis and precursor lesions	NPT: 0 Pancreatitis: N/A Precursor lesions: N/A PDAC: ++	[86,87]
	**GRP78**	*Neoplastic cells:* Co-Receptor for various proteins *Normal cells:* Chaperone protein localized in ER	Cell-to-cell and cell-to-matrix recognition processes, induction of endoplasmic reticulum stress for cell aging, survival, metastasis	Cell membrane, pancreatic neoplastic cells (in non-tumor cells located in ER)	N/A	Diffuse and high expression in PDAC Low expression in precursor lesions	Low expression in healthy pancreatic tissue No data available of expression profile in pancreatitis	NPT: − Pancreatitis: N/A Precursor lesions: − PDAC: ++	[90,149]
	**Integrin αvβ3**	Cell Adhesion Molecule	Tumor angiogenesis, tumor growth, metastasis	Cell membrane, (neoplastic) stromal and endothelial pancreatic cells	~68%	Diffuse, moderate expression in PDAC Moderate expression in precursor lesions	Low/moderate expression on healthy pancreatic tissue Moderate expression in pancreatitis	NPT: − to −/+ Pancreatitis −/+ Precursor lesions: −/+ PDAC: +/++	[93,97]
	**Integrin αvβ6**	Cell Adhesion Molecule	Tumor growth, metastasis	Cell membrane, (neoplastic) epithelial cells	80–88%	Diffuse, high expression in PDAC Moderate expression in precursor lesions	Low expression on healthy pancreatic tissue Moderate expression in pancreatitis	NPT: −/+ Pancreatitis −/+ Precursor lesions: −/+ PDAC: ++	[44,96,98]
	**Mesothelin**	GPI-anchored protein (Adhesion molecule)	Cell proliferation, migration, metastasis, inhibition of apoptosis	Cell membrane of pancreatic (neoplastic) mesothelial cells	>90%	Diffuse, high expression in PDAC No expression in healthy pancreatic tissue and in pancreatitis	Minimal expression in most precursor lesions	NPT: 0 Pancreatitis: 0 Precursor lesions: 0/− PDAC: ++	[102,103]
	**MT1-MMP/MMP-14**	Cell membrane associated enzyme	Tumor growth, invasiveness, resistance to chemotherapy	Cell membrane, (neoplastic) pancreatic stromal cells	~75%	Diffuse, high expression in PDAC Low expression in pancreatitis	Low expression in healthy pancreatic tissue Low expression in precursor lesions	NPT: − Pancreatitis: −/+ Precursor lesions: −/+ PDAC: ++	[108,150]
	**Mucin-1**	Protective cell coating	Cell proliferation, tumor invasiveness due to upregulated cell motility, metastasis	Cell membrane, (neoplastic) pancreatic epithelial cells	~90%	Diffuse, high expression in PDAC Low/moderate expression in precursor lesions	Low expression healthy pancreatic tissue No data available of expression profile in pancreatitis	NPT: − Pancreatitis: N/A Precursor lesions: −/+, + PDAC: ++	[112,151]
	**NTSR1**	G-protein-coupled Receptor (GPCR)	Cell proliferation, inhibition of apoptosis.	Cell membrane, (neoplastic) pancreatic cells	79–88%	Diffuse and high expression in PDAC Low expression in healthy pancreatic tissue	Low expression pancreatitis No data available of expression profile in precursor lesions	NPT: − Pancreatitis: + Precursor lesions: N/A PDAC: ++	[114,115]
	**PSMA**	Cell membrane associated enzyme	Tumor angiogenesis	Cell membrane, neovascular associated cells and tumor cells	~68%	Diffuse, moderate/high expression in PDAC No expression in healthy pancreatic tissue and in pancreatitis	No data available of expression profile precursor lesions	NPT: 0 Pancreatitis: 0 Precursor lesions: N/A PDAC: +/++	[119,120]
	**Tissue Factor (TF)**	Cytokine-receptor	Initiating blood coagulation cascades, metastasis	Cell membrane, (neoplastic) pancreatic cells	50–90%	Diffuse, high expression in PDAC Moderate/high expression in precursor lesions Low expression healthy pancreatic tissue	Low expression in pancreatitis	NPT: − Pancreatitis −/+ Precursor lesions: +/++ PDAC: ++	[127,128,129]
	**TfR1**	Ion-channel coupled Receptor	Cell proliferation, regulation of iron uptake/release.	Cell membrane, (neoplastic) pancreatic cells	>90%	Diffuse, high expression in PDAC No expression in healthy pancreatic tissue	No data available of expression profile in pancreatitis and precursor lesions	NPT: 0 Pancreatitis: N/A Precursor lesions: N/A PDAC: ++	[135,136]
	**uPAR/uPA system**	GPI-anchored receptor	Degradation of ECM, tumor angiogenesis, metastasis	Cell membrane, stromal (neoplastic) cells Cell membrane, endothelial (neoplastic) pancreatic cells	~80% ~67%	Diffuse, moderate expression PDAC and surrounding stroma Moderate expression in precursor lesions Low expression in healthy pancreatic tissue	Moderate/high expression in negative lymph nodes No data available of expression profile in pancreatitis	NPT: − Pancreatitis: N/A Precursor lesions: + PDAC: +/++	[44,138,140]
	**VEGFR-2/** **VEGF-A**	Tyrosine kinase Receptor (TKR) Growth factor	Tumor angiogenesis	Cell membrane, pancreatic vascular endothelial cells	>70%	Diffuse and high expression in PDAC Low expression in healthy pancreatic tissue	Low expression in pancreatitis No data available of expression profile in precursor lesions	NPT: − Pancreatitis: − Precursor lesions: N/A PDAC: ++	[143,152]

An overview is given of the key identified and evaluated biomarkers/tumor targets for molecular imaging in PDAC. Categorized in alphabetical order. Biological function, as opposed to carcinogenic effect related to expression on tumor (associated) cells of the pancreas. Positive target expression and main advantages/disadvantages for molecular imaging have been summarized based on expression profile in the normal pancreatic parenchyma, pancreatitis, pancreatic precursor lesions, and PDAC. The colors are related to the biological subtype or target, matched colors are targets of the same subtype. 0 = No expression; − = Low expression; + = Moderate expression; ++ = High expression; N/A = Not available/Unknown. Abbreviations: CAF = Cancer-associated fibroblast; Cath-E = Cathepsin-E; CA19.9 = Carbohydrate antigen19.9; CDCP1 = CUB domain-containing protein-1; CEA = Carcinoembryonic antigen; EGFR = Epidermoid growth factor receptor; EpCAM = Epithelial cell adhesion molecule; FAPα = Fibroblast activating protein-α; GRP78 = Glucose regulating protein-78; MMP = Matrix metalloproteinase; NTSR-1 = Neurotensin receptor-1; NPT = Normal healthy pancreatic parenchyma; PDAC = Pancreatic ductal adenocarcinoma; PSMA = Prostate membrane antigen; TfR1 = Transferrin receptor-1; TF = Tissue Factor; uPa = Urokinase-type plasminogen activator; uPAR = Urokinase-type plasminogen activator Receptor; VEGFR(2) = Vascular endothelial growth factor receptor; VEGF-α = Vascular endothelial growth factor α.

## 3. Positron Emission Tomography—Computed Tomography (PET/CT)

PET/CT has the unique ability to provide information on biochemical activity of cancer tissue. In the last decade, several PDAC-targeted PET-tracers have been evaluated in (pre)clinical studies for their potential to provide valuable diagnostic information in various stages of disease.

### 3.1. Primary Diagnostic Work-Up and Monitoring Response to Neoadjuvant Treatment

Currently, the diagnostic work-up of suspected pancreatic lesions is ce-CT. Current guidelines are indifferent on the role and diagnostic value of FDG-PET/CT [8]; however, this might change in the future based on recent literature as discussed in Section 4.2. Furthermore, with the implementation of neoadjuvant treatment schedules as standard of care for borderline resectable tumors, accurate treatment response monitoring and determination of resectability is important. As the current imaging methods are insufficient to predict response to NT, additional diagnostic tools are needed [32,33]. PET/CT could potentially contribute to this by visualizing and predicting changes in biochemical activity and tumor volume before and after NT. Treatment response monitoring using PET/CT has only been evaluated in clinical trials using radiolabeled glucose (^18^F-FDG), which is one of the few clinically approved PET-tracers. PDAC-targeted PET-radiotracers are currently still in the early phases of (pre)clinical development and research is mainly focused on feasibility and performance during primary diagnostic work-up. Until now, no reports have been published on the clinical use of molecular targeted PET-imaging for treatment response monitoring in PDAC.

### 3.2. Clinically Available PET-Tracers for PDAC Imaging

#### 3.2.1. ^18^F-FDG

^18^F-FDG is currently the most widely used PET-tracer in cancer imaging and shows uptake in tissues with increased metabolic activity such as cancer or infection. The major drawback of ^18^F-FDG PET/CT imaging for visualizing a suspected pancreatic mass is the low specificity for accurate differentiation between malignant, pre-existent, and inflammatory tissue. However, recent studies demonstrated superior sensitivity for detecting distant metastatic disease, especially in the liver, when compared to ce-CT [153,154,155]. The PET-PANC study, a large multicenter prospective study conducted in the United Kingdom, showed that in 11% of the patients with primary suspected pancreatic malignancies, the management and therapeutic strategy were changed based on PET/CT findings (including avoidance of futile surgeries in 20% of patients) [154]. Feasibility and performance of FDG-PET/CT for monitoring response in PDAC has been evaluated in several smaller trials with favorable results. Two prospective trials by Heinrich et al., and Zimmermann et al. together with three retrospective trials by Yokose et al., Dalah et al., and Panda et al. demonstrated that a decrease in ^18^F-FDG-uptake in PDAC was associated with a histopathologic response to treatment and a decrease in tumor size [36,37,156,157,158]. Yoshioka et al., showed that ^18^F-FDG-PET/CT could add valuable diagnostic information by demonstrating that a decrease in tumoral metabolic FDG uptake after NT tends to precede anatomic changes in tumor size as seen on ce-CT and MRI [159]. These results suggest that response to treatment might be visualized earlier using ^18^F-FDG-PET/CT than ce-CT and MRI. Following these results, additional prospective validation in larger studies is warranted and will possibly be provided by Dutch PANDIGIPET trial (NTR7442), which evaluated treatment response using digital ^18^F-FDG-PET/CT and compared the results to the initial CT scans. This trial was recently completed, and results are expected soon.

#### 3.2.2. ^18^F-Fluorothymidine (FLT)

^18^F-FLT is a radiotracer that accumulates in active proliferating cells and indicates the activity of the enzyme thymidine kinase. Although known as highly proliferative, uptake in PDAC was poor and significantly less compared to ^18^F-FDG, resulting in visualization of PDAC in only 40% of six included patients. Tracer activity in the primary tumor could be accurately differentiated from background tracer activity in only two patients. [160]. However, Wieder et al. suggest ^18^F-FLT-PET/CT might serve a purpose in determining prognosis and survival, as increased proliferative rates are associated with more aggressive tumors and decreased OS [161].

#### 3.2.3. ^18^F-Fluoromisonidazole (FMISO) and ^18^F-Fluoroazomycin Arabinoside (FAZA)

^18^F-FMISO and ^18^F-FAZA are hypoxia-based radiotracers which, despite the favorable hypoxic condition in PDAC, showed minimal uptake in PDAC. Segard et al. showed ^18^F-FMISO resulted in tumor-to-background ratio’s (TBR) ranging from 1.4 to 2.9. Yamane et al. used ^18^F-FMISO and identified in 9/25 (36%) PDAC patients the primary tumor, peak TBRs of the negative cases were significantly lower than that of the positive cases. In conclusion, no significant correlation between tumor perfusion and hypoxia, nor an association between tumor volume and hypoxia, was observed in three conducted studies. [162,163,164].

### 3.3. PDAC-Targeted PET-Tracers in Clinical Early Clinical Trials

PDAC-targeted radiotracers, which have been evaluated in early phase clinical trials, will be discussed in more detail, an overview of all clinical studies is depicted in Table A1.

#### 3.3.1. CA19.9

CA19.9 has been extensively evaluated as serum biomarker, since it is strongly correlated with disease stage. Therefore, using CA19.9 overexpression on PDAC cells was one of the early molecular targets of interest for targeted molecular imaging in PDAC. Lohrmann et al. demonstrated that ^89^Zr labeled anti-CA19.9 human antibody HuMab-5B1 (^89^Zr-MVT-2163) was able to detect the primary pancreatic tumor, metastases, and suspected lymph and unsuspected (additional) nodes in 12 patients with an in vivo TBR > 18 [165]. The relatively long optimal window between administration and imaging (7 days) makes application of CA19.9 for primary diagnosis less suitable. A solution would be using a radioligand with a shorter half-life time, such as fluor-18 (18F) and/or smaller molecular constructs such as peptide sequences. An interesting future application for CA19.9 targeted imaging was recently preclinically evaluated by Houghton et al. showing promising results for a dual-labeled anti-CA19.9 PET/fluorescence tracer (^89^Zr-^ss^dual-5B1), which could be used for pre-operative staging and navigation with PET/CT followed by intraoperative guidance with NIR-fluorescence [166].

#### 3.3.2. Fibroblast Activating Protein (FAP)

PET/CT imaging using radiolabeled FAP inhibitors (FAPI) has recently been investigated in PDAC patients by Kratochwil et al.; they demonstrated intermediate to high accumulation of ^68^Ga-FAPI-04 in 50 PDAC patients. With minimal accumulation in surrounding benign tissue (including the liver), this resulted in significant tumor uptake with a SUV_max_ ranging from 6 to 12 [83]. A second study retrospectively compared ^68^Ga-FAPI PET/CT to ce-CT in 19 patients with suspected PDAC, and found that the use of ^68^Ga-FAPI PET/CT resulted in detection of additional metastases and subsequent upstaging of 1 out of 7 primary PDAC patients and 8 out of 12 recurrent/progressive PDAC patients [167]. In conclusion, the use of ^68^Ga-FAPI PET/CT showed promising results to improve diagnosis and (re)staging of PDAC, and might in the future be combined with FAP-targeted therapies (e.g., radionuclide therapy), which has already been evaluated in the preclinical setting by Lui et al., demonstrating a significant tumor suppressing effect using ^177^Lu-FAPI-46 and ^225^Ac-FAPI-46 in PANC-1 xenograft mouse models. [167,168,169].

#### 3.3.3. Integrin αvβ6

Visualizing tumor associated neoangiogenesis using integrin αvβ6 targeted PET/CT has been evaluated in several smaller (pre)clinical trials [94,95,170]. The first clinical results by Kimura et al. show that their cystine knotted peptide-based PET-tracer (^18^F-FP-R_0_1-MG-F) is safe and resulted in high specific uptake in pancreatic lesions, with minimal background uptake in the liver [170]. Nakamoto et al., which published their preliminary results in an abstract, used a comparable integrin αvβ6 targeted tracer ^18^F-FP-R_0_1-MG-F2 in a cohort of 14 PDAC patients, and accurately detected all known PDAC as well as metastatic lesions, with high tumor uptake [171]. Feng et al. evaluated a cyclic αvβ6-targeting radiolabeled peptide (^68^Ga-cycratide) and showed high specificity for PDAC lesions, with low background signal in the surrounding organs [95]. Considering that the expression profile of αvβ6 is not altered after neoadjuvant therapy (FOLFIRINOX), evaluating αvβ6-targeted PET/CT for treatment response monitoring is of particular interest [31]. Summarizing, these trials with integrin αvβ6-targeted PET/CT showed significant sensitivity and specificity for accurate visualization of PDAC and its metastases. This warrants further evaluation in (multicenter) phase III trials for evaluation of the diagnostical value.

#### 3.3.4. PSMA

Tumor-targeted PSMA PET/CT has established a vital role in staging and determining treatment strategy in prostate cancer. In addition to prostate cancer, PSMA is expressed related to tumor-associated neovasculature and tumor cells in several other malignancies, including PDAC. Krishnaraju et al. published the first clinical trial including 40 patients with suspected pancreatic lesions. ^68^Ga-PSMA-11 PET/CT outperformed ^18^F-FDG PET/CT for detection of primary PDAC with increased sensitivity (94.7% vs. 89.5%), specificity (90.5% vs. 57.1%), negative predictive value (95% vs. 87.5%), and accuracy (92.5% vs. 72.5%). Furthermore, pancreatitis lesions mimicking PDAC showed false positive uptake on ^18^F-FDG PET/CT but showed no uptake on PSMA PET/CT [124]. Since PSMA expression in PDAC is not altered after neoadjuvant treatment (FOLFIRINOX), evaluating PSMA-targeted PET/CT for treatment response monitoring is of particular interest [31]. If successful, treatment might be combined with PSMA-targeted therapies (e.g., radionuclide therapy), which have already been investigated in prostate cancer [122,123].

### 3.4. Preclinical Evaluation and Development of PDAC-Targeted PET-Tracers

In addition to the previously mentioned radiotracers, there are several promising targets for PDAC targeted PET/CT or PET/MRI imaging currently evaluated in preclinical studies. These include integrin αvβ3, CDCP-1, EGFR, NTSR1, Tissue Factor (TF), and uPA/uPAR system. Details from all preclinical studies are depicted in Table A2.

Trajkovic-Arsic et al. developed a safe and effective RGD targeted peptide (^68^Ga-NODAGA-RGD) specifically targeting integrin αvβ3. Which was tested in an orthotopic PDAC mouse model, showing accurate identification of PDAC with TBRs >10 and relatively low background signal in the liver [172].

Moroz et al. evaluated their CDCP-1 targeted tracer (^89^Zr-DFO-4A06) and showed high specific tumor uptake and favorable rapid renal clearance in a subcutaneous PDAC mouse model [63].

Boyle et al. constructed an EGFR targeting F(ab′)-fragment (^64^Cu-panitumumab), which showed significant tumor and minimal background uptake in PDAC from two patient derived cell lines, resulting in clear tumor visualization [173].

Prignon et al. constructed a NTSR1 targeted peptide-based PET-tracer (^68^Ga-DOTA-NT-20.3) with PDAC TBRs >3.5 and low background in the liver and small intestines. They also showed that DOTA-NT-20.3 was able to distinguish PDAC from pancreatitis [116]. Hodolic et al. recently published the first clinical results from three PDAC patients, demonstrating identification of primary tumor in three out of three patients and distant metastatic disease in two out of two patients [118].

Nielsen et al. constructed and evaluated a TF targeted antibody PET-tracer (^64^Cu-NOTA-FVIIai) in a subcutaneous PDAC mouse model. PET/MRI imaging resulted in adequate visualization of PDAC with a tumor-to-muscle ratio (T/M ratio) >30, intermediate/high tracer uptake in the liver was observed [131].

Last, a uPAR-targeted antibody (^89^Zr-Df-ATN-291) was validated by Yang et al. in a subcutaneous tumor mouse model in various cancer types, including PDAC, resulting in favorable biodistribution and a tumor-to-muscle ratio >7 based on PET/CT-imaging 24 h after tracer administration [174].

### 3.5. Summary

In conclusion, previous research has shown FAPI, PSMA, integrins αvβ3/αvβ6, and CA19.9 to hold the most potential for tumor-targeted PET-imaging of PDAC. However, most of these tracers are still in the early phases of clinical development and larger studies are needed. Within our own research group, two clinical trials have been initiated evaluating the diagnostic performance of ^18^F-DCFPyL-(PSMA)-PET/CT (Netherlands Trial Register NL8919) targeting PSMA, which has been closed for inclusion and analysis of results is pending. Second, patients have currently been recruited in a clinical trial evaluating ^18^F-Fluciclatide-(RGD)-PET/CT targeting integrins αvβ3, αvβ5 and αvβ6 (Netherlands Trial Register NL7605). Progress has been made in recent years evaluating PDAC-targeted PET-tracers, and early phase clinical trials have shown some favorable results and added value, but more data is needed before PET/CT imaging will be integrated in the workflow of PDAC.

## 4. NIR-Fluorescence Imaging and Fluorescence-Guided Surgery

Over the last decade, image-guided surgery with NIR-fluorescence (NIRF) has been studied and developed for various malignancies [175,176]. With improved neoadjuvant treatment strategies more patients suffering from PDAC have become eligible for surgical treatment. Considering that surgery is moving towards minimally invasive techniques (i.e., laparoscopic, and robotic surgery), procedures are becoming more complex for the surgeon. Although minimally invasive surgery offers benefits for the patient such as smaller incisions and a shorter hospital stay compared to conventional open surgery, an important drawback is the loss of tactile feedback for the surgeon, potentially hampering accurate identification of tumor margins and vital structures adjacent to the tumor, of which iatrogenic damage must be avoided. Accurate tumor visualization and demarcation is pivotal for performing safe and complete surgical resections, which emphasizes the importance of additional tools to compensate for the loss of tactile feedback. Fluorescence-guided surgery (FGS) using NIRF imaging, can potentially fulfill this role by providing surgeons with real-time intraoperative fluorescence-enhanced tumor visualization [40].

NIRF imaging uses a NIR-fluorescent tracer that has been administered intravenously. Most fluorescently labeled tracers emit light in the NIR-spectrum, mainly in the NIR-I (650–900 nm) window; although, experimental studies have been conducted with tracers emitting in the NIR-II window (1000–1700 nm), latter technique is still in the experimental phase due to difficulties with fluorophore stability and toxicity [177]. For the imaging procedure, an external light source is required, which emits photons at a certain wavelength in order to penetrate tissue and reach the fluorescently labeled tracer bound to the target tissue. Upon entering and passing through tissues, these photons will be either absorbed by the fluorophore, reflected by the surface of imaged tissue or refracted. Absorption leads to excitation of the fluorophore, which induces emission of NIR-light of a longer wavelength. This difference in peak excitation and emission wavelength is referred to as the Stokes shift [40]. A NIR-camera system transforms the emitted signal in real-time to fluorescent images that are projected on a monitor in the operation room [178]. NIRF-tracers can roughly be divided into two categories: untargeted tracers that predominantly enhance lesions or structures by the enhanced permeability and retention effect (EPR effect) or perfusion (Indocyanine Green, *ICG*, 800 nm or methylene blue, *MB*, 700 nm), and targeted tracers that consist of a target-specific molecular probe conjugated to a fluorescent dye [179]. Until this moment, only ICG and MB are FDA approved for intraoperative NIR-fluorescence imaging. Although ICG is an effective untargeted agent to visualize biliary structures during pancreaticobiliary reconstructions in order to avoid iatrogenic damage, the currently known untargeted fluorescent agents have demonstrated limited applicability for adequate tumor visualization during cancer surgery [180,181,182]. Therefore, the main focus in FGS (pancreatic) cancer surgery in recent years is identification and synthesis of effective and safe tumor-specific NIRF-tracers, which enable enhanced visualization of the primary tumor, metastasized lymph nodes, and distant metastases.

### 4.1. Clinically Tested PDAC Targeted NIRF-Tracers

The following sections will review several conducted clinical studies evaluating tumor-targeted NIRF-imaging in PDAC. More detailed information regarding these studies is presented in Table A1.

#### 4.1.1. CEA

After successful preclinical testing of fluorescent CEA-antibody constructs [66,70,183], Hoogstins et al. performed the first in-human dose-escalation trial using SGM-101, a chimeric antibody to CEA conjugated to the fluorophore BM-104 (700 nm). In 11 patients with resectable PDAC, NIR-imaging identified all primary pancreatic tumors and metastatic lesions (3/3). A moderate level of autofluorescence of the background tissue was observed, probably related to the 700 nm wavelength, resulting in calculated TBRs of, respectively, 1.6 (tumor) and 1.7 (metastases) [68].

#### 4.1.2. EGFR

EGFR-targeted NIRF imaging for PDAC has been evaluated in two clinical trials, with promising results. Preclinical research was conducted primarily in head and neck cancers [184]. Tummers et al. performed phase II clinical trial evaluating cetuximab conjugated to IRDye800CW (800 nm) in non-pretreated patients; intraoperative imaging was performed 2–5 days after administration. This study reported accurate identification of all tumors and metastatic lesions with a sensitivity of 96.1% and specificity of 67.0%. NIRF-guided resection of the tumor and/or metastatic lymph nodes (LN) was possible in four out of six patients with in vivo TBRs of, respectively, 2.3 and >6 [185]. Mean fluorescence intensity (MFI) was significantly different between PDAC, normal pancreatic parenchyma, and pancreatitis. This finding is particularly interesting since pancreatitis is known for its PDAC mimicking appearance on radiological imaging [185]. Tummers et al. used this same construct for evaluation of the feasibility for the detection of visually occult metastatic lymph nodes by ex vivo NIRF imaging. Cetuximab-IRDye800CW was able to accurately identify metastatic LN’s macroscopically with a sensitivity and specificity of, respectively, 100% and 78%, as well as occult tumor deposits (<5 mm) with a sensitivity of 88%. Although these results are from ex vivo imaging, it shows proof for fluorescence identification of nodal metastases, which were not previously seen with CT-imaging [186]. Lu et al. performed a phase I trial, evaluating panitumumab-IRDye800CW in 11 non pretreated PDAC patients. Intra-operative NIRF imaging was performed 2–5 days after administration, resulting in identification of the primary tumors, metastatic LNs, and small (<2 mm) peritoneal metastasis in all patients. With adequate TBRs of >3 in the 50 mg dosing cohort. Furthermore, fluorescence signal delineating the tumor correlated with histopathology in all cases and significant higher MFIs in LN+ than LN- were observed [187].

#### 4.1.3. VEGF-A

VEGF-A targeted NIR-imaging using bevacuzimab-IRDye800CW, a monoclonal anti-VEGF antibody conjugated to IRDye800CW (800 nm), has been described in patients with breast cancer as well as with colorectal peritoneal metastases. [188,189] Our medical center, together with University Medical Center Groningen (UMCG) performed a phase II feasibility trial in patients with pancreatic tumors. Although, intraoperative tumor identification was feasible and administration of bevacuzimab-IRDye800CW was safe, the combination of moderate TBRs and indifferent fluorescence signals in residual healthy pancreatic parenchyma resulted in early termination of this study, further analyses are pending (NCT02743975).

### 4.2. Preclinical Evaluation and Development of PDAC-Targeted NIRF-Tracers

All clinically evaluated tracers to date are antibody-based, which may be less able to penetrate the dense PDAC stroma, compared to smaller-sized carrier molecules (e.g., peptides, Fab’-fragments, nanobodies, nanoparticles, and DARPins (Designed Ankyrin Repeat Proteins)) [45,46]. As suboptimal penetration could result in a suboptimal target binding and, consequently, an insufficient fluorescence signal. Development and evaluation of smaller-sized carriers, which, due to their size, will be less influenced by the TME, potentially possess superior and faster tumor penetration, resulting in improved signal intensity. Furthermore, a smaller interval between tracer-administration and imaging is likely to be achieved by using smaller-sized carriers. This section focuses on promising preclinical studies evaluating PDAC-targeted NIRF-tracers, of which the preclinical results might warrant future clinical translation. These include the following targets: CA19.9, integrin αvβ3/αvβ5/αvβ6, NTSR1, and the uPA/uPAR system. An overview of all preclinical studies evaluating PDAC-targeted NIRF-imaging are listed in Table A2.

Houghton et al. constructed a dual-labeled anti-CA19.9 PET/Fluorescence tracer (^89^Zr-^ss^dual-5B1), which was tested in a subcutaneous mouse model using small-animal PET/CT-imaging, followed fluorescence-guided resected with NIRF imaging [166]. Lwin et al. evaluated for the first time a humanized CEA-targeted antibody conjugated to a NIR-fluorophore in an orthotopic PDAC tumor mouse model showing accurate tumor identification with TBRs >16. This humanized antibody has already been tested in clinical trials in head and neck cancer [66,184].

Integrin αvβ3/αvβ5/αvβ6 targeting using a cyclic configured peptide, cRGD-ZW800-1 (800 nm), performed by Handgraaf et al., has shown some promising results in a preclinical study in orthotopic mouse models of breast, colorectal, and pancreatic cancer cell lines [190]. The first clinical trial using cRGD-ZW800-1 in healthy volunteers and patients with colorectal carcinoma was initiated shortly after these findings. cRGD-ZW800-1 was safe and allowed accurate NIRF imaging and intra-operative delineation of the colorectal tumors 2 h after intravenous (IV) administration, with TBRs >1.5 in the highest dosing cohort (0.05 mg/kg) [191]. Specific integrin targeting for αvβ6 has been evaluated by Tummers et al., and they evaluated the cysteine knottin peptide R_0_1-MG, conjugated to IRDye800CW (800 nm). They showed specific αvβ6 binding and fluorescence signal in PDAC tumors in an orthotopic mouse model, TBRs ranged from 1.2 to 3.6; furthermore, using R_0_1-MG-IRDye800CW, successful distinction between PDAC and peritumoral inflammation could be made [192]. Based on the >90% overexpression of integrin αvβ6 in PDAC combined with the limited affected expression profile after neoadjuvant therapy, targeting αvβ6 using this construct potentially has the ‘ideal’ characteristics for fluorescence imaging of PDAC after neoadjuvant treatment [31,192].

Yin et al. performed a study in a subcutaneous/orthotopic PDAC tumor mouse using their NTSR1 receptor-targeted linear peptide NT for construction of a targeted PET-tracer (^64^Cu-AmBaSar-NT), as well as a NIRF-tracer (IRDye800-NT, 800 nm). This resulted in adequate visualization of orthotopic mouse PDAC tumors against low background of the liver and small intestine, with TBRs of 8.1 and 6.7 after 30 min and 1 h, respectively [115]. This demonstrates the potential advantages of smaller peptide structures over antibodies for clinical application, as shown by the short time between administration and imaging.

Juhl et al. constructed a uPA/uPAR system using a targeted linear peptide (AE105) conjugated to ICG (800 nm). They evaluated Glu-Glu-AE105-ICG in an orthotopic PDAC tumor mouse model, for identification of the primary pancreatic tumor and metastases 6–24 h after IV administration, resulting in TBRs in the primary tumor of 3.5 and in metastases of 3.4. Furthermore, they were able to identify and resect six additional metastases (14%), not visualized with white light inspection (WLI), as small as 1 mm in four out of eight mice, using NIRF imaging guidance [193]. The abovementioned pre-clinical studies show some promising results, and clinical translation is necessary to assess the safety, performance, and value of these PDAC targeted imaging agents in the clinic.

### 4.3. Summary

The field of targeted NIRF imaging is expanding rapidly as demonstrated by the abovementioned studies and the search for suitable PDAC-targeted NIRF-tracers continues. In conclusion, three targets have been evaluated in smaller clinical pilot studies, of which EGFR-targeted NIRF imaging with cetuximab-IRDye800CW or panitumumab-IRDye800CW demonstrated the most clinically relevant results, which warrants further evaluation in future trials for determination of the true impact on clinical/surgical management. CEA-targeted NIRF imaging using SGM-101 was feasible, although insufficient contrast in fluorescent signals (TBR’s < 2) could hamper adequate tumor delineation and assessment of resection margins. Based on the limited available information, VEGF-A-targeted bevacuzimab-IRDye800CW demonstrated tumor visualization; although, it has proven insufficient for accurate tumoral delineation and real-time assessment of the resection margins. An important factor that needs to be taken into consideration when evaluating clinical suitability of current and future NIRF-tracers, is the potential influence of NT on the TME composition, which is currently underexplored due to the inclusion of only non-pretreated patients. As demonstrated on neoadjuvant treated resected tissue specimens by Vuijk et al. [31], differential expression of targets was observed, which could negatively influence the intensity of tumor signal and related TBRs. Based on available literature, peptide- and Fab’-based tracers are closest to clinical evaluation [173,190,192], whereas smaller-sized nanobodies and nanoparticles are still in the early preclinical research phase [61].

## 5. Future Perspectives: Application of Improved Targeting Strategies, Technological Advances, and a Theranostic Approach

Molecular targeting enables visualization of tissue-specific biomarkers present within or around tumor cells. Ideally, these targets possess the characteristics suitable for molecular imaging during different stages of disease: early detection, treatment response monitoring, (re)staging, intra-operative guidance, as well as surveillance and follow-up. Interesting concepts aiming to improve the clinical value and applicability of (PDAC-) targeted molecular imaging are *dual-labeled* PET/NIRF-tracers, *dual-targeted*-tracers for PET/CT or NIRF imaging, cocktails of two or more separate tumor-targeted tracers, as well as application of *pre-targeting* strategies to improve tumor-specific contrast. A dual-labeled PET/NIRF-tracer has the potential to aid in pre-operative PET/CT (re)staging and the consecutive surgery by means of fluorescence guidance using a single systemic administration. Although, careful planning and choice of tracer combination based on half-life is important for effectively using dual-labeled tracers. Several dual-labeled PET/NIRF tracers have been described [166,194]. One example is the PDAC-targeted dual-labeled CA19.9 agent that has been evaluated in a preclinical stetting by Houghton et al. (Table A2), which demonstrated clear tumor visualization using small-animal PET/CT and NIRF guided resection of subcutaneous tumors in mice. [166]. Moreover, a dual-targeted tracer, which simultaneously targets two different targets within a heterogeneous tumor, or which targets two differentially expressed targets in heterogeneous patient populations, could enable improved sensitivity for tumor detectability [195]. An interesting strategy for application for PDAC could be a dual-labeled tracer targeting epithelial as well as stromal tissue. Within the preclinical field of PDAC research, Wang et al. (Table A2), have developed a bispecific NIRF-diabody (bi50-IRdye800) targeting VEGF-A/EGFR, which compared to uni-specific VEGF-A or EGFR single-chain variable fragments (scFv) resulted in improved clear delineation of PDAC lesions in a mouse model in vasculature rich areas (VEGF overexpression) as well the parenchymal rich areas (EGFR overexpression) [196]. Furthermore, Luo et al. (Table A2) developed a dual-targeted PET/CT tracer, targeting TF and endoglin, which demonstrated a clear tumor visualization with a (orthotopic) tumor-to-muscle ratio >70 [197]. Additionally, for dual-targeted imaging, providing a cocktail of two or more separate tumor-targeted tracers is a strategy to improve tumor-signals, on the other hand targeting more than one target potentially results in more false-positive signals. Lastly, a strategy to enhance visual contrast and reduce time between tracer administration and imaging is pre-targeting. Pre-targeting is based on separate administration of the targeting vector and radioligand or NIRF-fluorophore, aiming to decrease the circulation time of the radioligand/fluorophore, reduce its uptake in healthy nontarget tissues, which in case of PET/CT enabled enabling developing tracers with shorter half-life rates [198,199]. Houghton et al. developed a CA19.9-targeted PET tracer, and demonstrated adequate tumor delineation in a mouse model with a >25-fold reduction in total body radiation exposure compared to the non-pre-targeted control group [200].

The TME of pancreatic tumors contains biological barriers which makes targeting these tumors, for either imaging or therapy, challenging [201,202]. Using smaller (molecularly) sized carriers could be a solution to this problem. The preclinical field focusses on development and validation of smaller-sized carrier based tracers: peptides, Fab’-fragments, nanobodies [203], nanoparticles [204,205], and DARPins [206,207]. Advantages over these in comparison to mAbs are a reduced immunogenic response and most are renally cleared, as opposed to hepatic clearance [46]. Although, some developmental challenges related to these smaller-sized carriers, such as molecular stability, effective carrier extravasation and possibly inferior binding affinity must be considered, these compounds show promise for application in future PDAC-targeted molecular imaging and therapy [203,204].

Photoacoustic imaging (PAI) is an interesting technique in development for molecular imaging. PAI is based on the photoacoustic effect that is exhibited by several NIRF dyes, including 800CW or endogenous (tumor) tissue [208]. The photoacoustic effect is based on the principle of acoustic wave formation following light absorption caused by transient thermoelastic expansion of the target tissue. These acoustic waves are detected and analyzed by ultrasonic transducers to produce 2D or 3D images. PAI compared to NIRF has an improved optical contrast and spatial resolution with increased tissue penetration depth (up to 5 cm) [34,209]. While NIRF-imaging is suitable for detection of superficial lesions and assessment of surgical margins, PAI could be of complementary interest for deeper-seated tumors. Multimodal application of NIRF/PAI is an interesting future aspect, since both could use the same NIRF-dye. Tummers et al. demonstrated the applicability of PAI in a clinical trial using cetuximab-IRDy800 with multimodal molecular imaging including ex vivo PAI, resulting in an adequate photoacoustic signal of the tumor [185]. Available literature is scarce, since limited clinical or commercially available intra-operative systems are currently available [210].

In addition to diagnostic purposes, the advantages of tumor-targeting have also found their way to anti-cancer therapies and precision therapies, labelled as *theranostics* [211]. By substituting the radioisotope of targeted PET-tracer, the same vector molecule can be used for radionuclide treatment. Theranostics have opened new opportunities for the management of malignant lymphomas and various solid tumors, including PDAC. Various targeted PET-tracers can be conjugated to therapeutic radioisotopes, such as an α-emitter (^225^Actinium and ^213^Bismuth) or β-emitter (^177^Lutatium and ^90^Yttrium). An in-depth review of the principles and current developments of theranostics in PDAC was recently published by Montemagno et al., which provides a clear overview of the current advances in theranostics for diagnosis and treatment of PDAC [212]. An example includes the preclinical evaluation of the anti-FAP targeting vehicle FAPI labeled with ^225^Actinium in a PDAC mouse model (^225^Ac-FAPI) by Watabe et al., which demonstrated a significant tumor growth suppression after 3 weeks compared to control mice [213]. Theranostics have already shown promising (pre)clinical results regarding precision medicine and hold potential for application in and further improvement of therapeutic management of PDAC.

## 6. Conclusions

Molecular imaging of PDAC using PET and NIRF-probes can provide valuable information on tumor location during preoperative work-up and in real-time during intraoperative visualization and demarcation (Figure 1), thereby providing pre-, peri-, and postoperative clinical decision making. Identification and evaluation of several potent PDAC targets has led to the development and evaluation of multiple targeted PET/CT and NIRF-tracers, which, as shown by the wide variety of preclinical evaluated PDAC imaging agents, demonstrate the lack of one clear biomarker or target suitable for PDAC imaging. The value of tumor targeted PET/CT, NIRF-imaging or both has proven their diagnostic or therapeutic efficacy in early phase studies, including several clinical trials with small patient cohorts. Further refinement and advances in tracer development could result in dual specific and/or multimodal tracers that can be employed for various diagnostic and therapeutic modalities, including NIRF-imaging, FGS, PET/CT, and PAI. Interesting opportunities lie in the theranostic field, where targeted probes can be used to treat cancer by highly specific drug delivery to the tumor targets, and to visualize these targets by PET/CT imaging, which allows for accurate patient selection, pre- and post-treatment dosimetry, monitoring of therapy efficacy, and tumor (re)staging. Integration of targeted molecular imaging in the diagnostic and therapeutic management of PDAC has the potential to contribute to improved patient outcome and survival.

## Abbreviations

CAF	Cancer-associated fibroblast
CAM	Cell adhesion molecule
Cath-E	Cathepsin-E
CA19.9	Carbohydrate antigen 19.9
CDCP1 CUB	domain-containing protein1
Ce-CT	Contrast-enhanced Computed Tomography
CEA	Carcinoembryonic antigen
EGFR	Epidermoid growth factor receptor
EpCAM	Epithelial cell adhesion molecule
ESMO	European Society of Medical Oncology
FAP	Fibroblast-activating protein
FAPI	Fibroblast-activating protein inhibitor
FAZA	Fluoroazomycin arabinoside
FDG	Fluorodeoxyglucose
FGS	Fluorescence-guided surgery
FLT	Fluorothymidine
FMISO	Fluoromisonidazole
GRP78	Glucose-regulating protein-78
LAPC	Locally-advanced pancreatic cancer
mAb	Monoclonal antibody
MMP	Matrix metalloproteinase
MRI	Magnetic resonance imaging
NCCN	National Comprehensive Cancer Network
NET	Neuro endocrine tumor
NIR	Near infrared
NIRF	Near infrared fluorescence
NPT	Normal pancreatic tissue
NT	Neoadjuvant therapy
NTS	Neurotensin
NTSR-1	Neurotensin receptor-1
OS	Overall survival
PDAC	Pancreatic ductal adenocarcinoma
PSMA	Prostate membrane antigen
PET	Positron emission tomography
scFv	Single-chain variable fragment
SPECT	Single-photon emission computed tomography
SMI	Small molecule inhibitor
SUV	Standardized uptake value
TfR1	Transferrin receptor-1
TBR	Tumor-to-background ratio
TF	Tissue Factor
uPA	Urokinase-type plasminogen activator
uPAR	Urokinase-type plasminogen activator Receptor
VEGFR	Vascular endothelial growth factor receptor
VEGF-A	Vascular endothelial growth factor A
WLI	White light inspection

## Appendix A

**Table A1:** Clinical studies evaluating targeted molecular imaging (PET/CT—fluorescence Imaging) of PDAC.

Target	Tracer	Type	Modality (Control)	Study Design	Number of Patients	Infusion- Imaging Window	Main Outcome (Time-Point)	Results		Highlights	Main Elimination Route	Ref.
**CA19.9**	^89^Zr-DFO-Hu Mab-5B1	mAb, fully human	PET/CT (ce-CT)	Prospective (Phase II)	12 patients with local ized PDAC (LAPC)	After 24 h, 3 d, 4 d, 7 d	SUV_max_ (SD)	PDAC 24h: 3.51 (±2.58) PDAC Day 3: 9.3 (±14.3) PDAC Day 4: 12.6 (±14.5) PDAC Day 7: 16.5 (±17.3)		First in-human clinical study, high target affinity of CA19.9+ (PDAC) tumors. Location of tracer uptake on the imaging studies should allow for differentiation of PDAC and other malignan cies. Limitation: No histopatho logical confirmation of LN, Ideal injection-imaging win dow long (7 days), disad vantage for screening and early detection of PDAC, relatively high radiation ex posure.	Hepatic system	[165]
							In vivo Tumor-to-background ratio (SD)	18.4 (±1) 12/12 patients, at least one additional suspect metastatic lesion was identified.				
**CEA**	SGM-101-BM-104	mAb, chimiric	NIRF- 700 nm (−)	Prospective (Phase II)	12 Resectable PDAC patients (5, 7.5, 10 mg)	After 48 h	Identified pri mary tumors with NIRF	11/11 (100%), one surgical procedure abandoned before imaging		Proof-of-concept and safety targeting CEA with SMG101 for NIR-imaging of PDAC, metastatic lymph nodes, and distant metasta sis.	Hepatic system	[68]
							In vivo Tumor-to-background ratio (SD)	1.6 (±0.37)				
							In vivo Metas tasis-to-back ground ratio (SD)	1.7 (±0.42)				
**EGFR**	panitumumab-IRDye800CW	mAb, chimeric	NIRF- 800 nm (−)	Prospective (Phase I)	11 PDAC patients	After 2–5 days	Tumor-to-background ratio per dosage (SD)	25 mg: 3.0 (±0.5) 50 mg: 4.0 (±0.6) 75 mg: 3.7 (±0.4)		Proof-of-concept and safety of targeting EGFR with 50 mg panitumumab-IRDye800CW is best suita ble for NIR-imaging of PDAC, metastatic lymph nodes, and distant metasta sis.	Hepatic system	[187]
							Sensitivity Specificity (95% CI)	90.3% (84.5–94.2) 74.5% (65.1–82.1)				
							Ex vivo Differ entiating tumor from normal pancreatic parenchyma	Fluorescence signal delineat ing tumor correlated with his topathology in all cases MFI and the tumor-to-back ground ratio of the +LN were significantly higher than those of -LN (*p* < 0.001)				
	cetuximab-IRDye800CW, monoclonal antibody	mAb, chimeric	NIRF- 800 nm (−)	Prospective (Phase I/II)	7 Pancreatic tumors (5 PDAC, 2 NET)	After 2–5 days	NIRF Identifi cation of primary tumor	4/6 patients (67%)		Proof-of-concept and safety of targeting EGFR with 50 mg cetuximab-IRDye800CW is best suita ble for NIR-imaging of pan creatic tumors, metastatic lymph nodes, and distant metastasis. Potential to dif ferentiate pancreatitis and PDAC.	Hepatic system	[185,186]
							In vivo Tumor-to-background ratio (50 mg)	Primary Tumor: 2.3 (±0.72) Tumor+ LN: 6.3 (±0.82)				
							Ex vivo Tumor-to-back ground ratio (50 mg)	Primary Tumor: 3.4 (±0.4)				
							Sensitivity Specificity (95% CI)	96.1% (92.2–98.4%) 67.0% (59.7–73.8%)				
**FAP**	^68^Ga-FAPI-04	SMI	PET/CT (−)	Prospective, retrospectively analyzed (Phase II)	51 PDAC patients	After 1 h	SUV_max_	PDAC: 6–12 (range) Blood pool: 1.4 Muscle: 1.0		High FAPI uptake in FAP+ PDAC. Low background healthy tissues, including liver, resulting in moderate/high TBR’s in PDAC. Due to fast tracer uptake and clearance, optimal win dow for imaging: 10 min^−1^ h after injection, results in re duced radiation doses.	Renal System	[83]
	^68^Ga-FAPI-04, ^68^Ga-FAPI-46	SMI	PET/CT (ce-CT)	Prospective, retrospect-tive analysis (Phase II)	19 PDAC patients 7 primary; 12 progres-sive or recurrent disease	After 1 h	SUV_max_ (SD)	Pancreatitis: 7.50 (±3.52) PDAC: 13.37 (±5.45) Metastatic Lymph nodes: 14.13 (±8.50) Distant metastases: 7.34 (±2.48) Blood pool: 2.3 (8.31) Muscle: 2.4 (8.72)		High FAPI uptake in pri mary FAP+ PDAC, lymph nodes, distant metastases. Low background healthy tissues, including liver, re sulting in adequate TBR’s for PDAC. Differentiation with pancreatitis challeng ing. Clinical value should be ad dressed separately in ho mogenous group for pri mary diagnosis and ability of response monitoring.	Renal System	[167]
								Restaging in 10 out of 19 patients compared to ceCT				
							SUV_max_ (Tumor-to-background ratio)	Low hepatic background (SUV 1.7, compared to FDG-PET/CT (SUV 2.8)				
**Integrin αvβ6**	^18^F-FP-R_0_1-MG-F2	Peptide, cyclic	PET/CT (ce-CT)	Prospective (Phase I)	14 PDAC patients	After 1 h	SUV_max_ (categories)	PDAC: high Pancreas: moderate/high		Proof-of-concept and safety for PET-imaging with high specific affinity for αvβ6+ PDAC and metastatic le sions.	Renal System	[171] *
							Detection of known PDAC lesions	14/14 (100%)				
	^18^F-FP-R_0_1-MG-F	Peptide, cyclic	PET/CT (FDG-PET/CT)	Prospective pre-clinical/clinical study (Phase I/II)	10 Healthy volunteers 1 PDAC patient	After 1 h	Healthy volun teers: SUV_mean_	Liver: <1 Pancreas: 2 Muscle: 1.5 Stomach: 10 Small Intestines: 9		Proof-of-concept and safety for αvβ6+ targeted PET/CT-imaging in healthy volunteers and 1 PDAC patient. Compared to FDG-PET/CT lower SUVmean in surrounding/adjacent structures of the pancreas, except the stomach. Clinical evaluation in larger cohort of PDAC patients warranted (Nakamoto et al. above) Primary diagnosis as well as response monitoring could be evaluated.	Renal System	[170]
						After 1 h	PDAC patient SUV_mean_	αvβ6-PET/CT Liver: <1 PDAC: 6.2 Muscle: 1.8 Stomach: 22 Small Intestines: 9	FDG/PET/CT Liver: 2.9 PDAC: 4.1 Muscle: <1 Stomach: <1			
**MSLN**	^89^Zr-MMOT0530A	mAb, humanized	PET/CT (ce-CT)	Prospective (Phase I/II)	11 patients total 7 PDAC 4 Ovarian	After 2, 4, and 7 days	SUV_max_ (SD) (Day 4)	PDAC: 11.5 (±5.6) Metastases: 12.1 (±6.0) Muscle: 2.4 (±1.3)		MSLN was able to visualize PDAC, although high varia bility between SUVmax in PDAC patients. Resulting in relatively poor TBR’s. Optimal injection-imaging window 4 days. High up take in liver, possibly inter fering with surgical field of view. Potentially interesting to evaluate/predict response to MSLN targeted therapy	Hepatic system	[214]
							Tumor-to-back ground ratio	Day 1: 0.70 Day 4: 1.1 Day 7: 1.28				
								2 MSLN+ lung nodules missed on MSLN-PET/CT which were seen on ce-CT				
**NTSR1**	^68^Ga-DOTA-NT-20.3	Peptide, linear	PET/CT (−)	Prospective (Phase I)	3 patients localized or metastatic PDAC	After 5–25 min, 25–45 min, 45–65 min, and 65–85 min	Uptake in primary tu mors, meta static disease	Primary tumor: 3/3 patients Metastases: 2/2 patients		Proof-of-concept for safety and tolerability of ^68^Ga-DOTA-NT-20.3 in patients with proven localized or metastatic PDAC. Uptake of NT-20.3 uptake in all PDAC and in 2/3 patients with liver metastases.	Renal System	[118]
**PSMA**	^68^Ga-PSMA-11	Peptide, linear	PET/CT (FDG-PET/CT)	Prospective (Phase II)	19 PDAC 21 Benign pancreatic lesions	After 1 h	SUV_max_ (IQR)	PSMA- PET/CT:	FDG- PET/CT:	PSMA-PET/CT out-performed FDG-PET/CT in primary diagnosis of PSMA+ PDAC lesions. Lesions with inflammatory origin were not visualized with PSMA-PET/CT, in contrast to FDG-PET/CT (false positive). Promising results for PSMA-PET/CT, further evaluation in a multicenter study will be able to substantiate the diagnostic value of PSMA-PET/CT in the primary diagnosis of PDAC.	Renal System	[124]
								Benign: 3.9 (3.4) Malignant: 7.6 (10.8)	Benign: 3.5 (1.5) Malignant: 7.4 (4.5)			
							Sensitivity Specificity PPV NPV Accuracy	94.7% 90.5% 90% 95% 92.5%	89.5% 57.1% 65.4% 87.5% 72.5%			
**VEGF-A**	Bevacizu-mabIRDye 800CW, monoclonal antibody	mAb, chime-ric	NIRF- 800 nm (−)	Prospective (Phase II)	10 suspected pancreatic tumors (PDAC, NET, Periampul-lary, IPMN)	After 3 days	Detailed results have not yet been published			Bevacizumab-800CW was safe without adverse events related to the study drug although due to residual in different fluorescent signals in non-tumoral tissue after complete tumor resection in the majority of included pa tients, this study was termi nated early. Feasibility for further clini cal translation of VEGF-tar geted FGS of PDAC is based on these results not proven.	Hepatic system	Eudra-CT 2015-004247-39

An overview is given of clinically evaluated PDAC targeted PET/NIRF imaging agents, categorized in alphabetical order. Main study design features, results, and highlights are shown. Abbreviations: CA19.9 = Carbohydrate antigen 19.9; CDCP1 = CUB domain-containing protein-1; CEA = Carcinoembryonic antigen; EGFR = Epidermoid growth factor receptor; EpCAM = Epithelial cell adhesion molecule; FAPα = Fibroblast-activating protein-α; FGS = Fluorescence-guided surgery; GRP78 = Glucose-regulating protein-78; IPMN = Intraductal papillary mucinous neoplasm; IQR = Inter quartile range; LN = Lymph node; mAb = Monoclonal antibody; MMP = Matrix metalloproteinase; NET = Neuro endocrine tumor; NIRF = Near-infrared Fluorescence; NPV = Negative predictive value; NTSR-1 = Neurotensin receptor-1; PDAC = Pancreatic ductal adenocarcinoma; PPV = Positive predictive value; PSMA = Prostate membrane antigen; SD = Standard deviation; SMI = Small Molecule Inhibitor; SUV = Standardized uptake value; TBR = Tumor-to-background ratio; TfR1 = Transferrin receptor-1; TF = Tissue Factor; uPa = Urokinase-type plasminogen activator; uPAR = Urokinase-type plasminogen activator Receptor; VEGFR(2) = Vascular endothelial growth factor receptor; VEGF-α = Vascular endothelial growth factor α. * Only abstract available.

**Table A2:** Pre-clinical studies evaluating targeted molecular imaging (PET/CT—fluorescence Imaging) of PDAC.

Target	Tracer	Type	Modality	Design	Subjects (Cell Line)	Infusion-Imaging Window	Main Outcome	Results	Highlights	Ref.
**CA19.9**	Alexa Fluor 488-anti-CA19.9	mAb, hu manized	Fluorescence imaging- 500 nm	In vitro/In vivo Preclinical probe construction and target validation in mouse model	Orthotopic PDAC mouse model (CFPAC, BxPC-3, PANC-1)	After 24 h	Ability to visualize CA19.9+ PDAC after 24 h	Small tumors were virtually uni dentifiable under standard bright-field imaging but were clearly visible using fluorescence imaging. Administration of AlexaFluor 488-anti-CA19.9 facilitated visualization of experimental metastatic implants in the spleen, liver, and peritoneum at laparotomy. All metastatic lesions in the spleen, liver, and peritoneum were confirmed by histologic evaluation following whole-body imaging.	Proof-of-concept of in vivo fluorescence imaging of CA19.9+ PDAC with fluorescence imaging. Low expression/fluorescence on surrounding stromal tissue. Low background due to low CA19.9 expression in normal tissue. Additional evaluation is war ranted to address imaging characteristics and validate fluorescence signals.	[215]
	^124^I-anti-CA19-9	Diabody	PET/CT	In vitro/In vivo Preclinical probe construction and target validation in mouse model	Subcutaneous PDAC mouse model (BxPC3: CA19.9+ Capan-2: CA19.9+ MiaPaCa-2: Ca19.9−)	After 4 h and 20 h	Tumor-to-background ratio (blood pool)	All cell lines: 3.0 BxPC3: 5.0 Capan-2: 2.0 BxPC3: 11.0	Proof-of-concept PET/CT-imaging of CA19.9+ PDAC. The cys-diabody demonstrates target-specific binding of human pancreatic cancer cells allowing tumor visualization, and with the potential to deliver targeted treatment. Relative high uptake in the liver, which could interfere with imaging of the pancreas.	[216]
							Positive-to-negative tumor ratio	Capan-2: 6.0 BxPC3 (tumor+): 1.1 (0.5–1.8)		
							Tumor uptake/biodistribution in % of injected dosage/g BW (range)	BxPC3 (tumor−): 0.1 (0.03–0.2) Capan-2 (tumor+): 0.5 (0.3–0.1) Capan-2 (tumor−): 0.1 (0.06–0.1)		
	^124^I-anti-CA19-9	Cysteine-modified diabody	PET/CT	In vitro/In vivo Preclinical probe construction and target validation in mouse model	Subcutaneous PDAC mouse model (BxPC3: CA19.9+ MiaPaCa-2: Ca19.9-)	After 4 h and 20 h	Tumor-to-blood ratio	BxPC3: 2.7 BxPC3: 6.0	Proof-of-concept PET/CT-imaging of CA19.9+ PDAC. High target binding affinity for CA19.9 allowing tumor visualization. Shorter half-life-time than diabody in comparison with study from Girgis et al. above.	[217]
							Positive-to-negative tumor ratio	BxPC3 (tumor+): 1.1 (0.4–1.7)		
							Tumor uptake/biodistribution in % of injected dosage/g BW (range)	BxPC3 (tumor−): 0.2 (0.1–0.3)		
	^89^Zr(ss)DFO-5B1	mAb, fully hu man	PET/CT, NIRF- 800 nm (dual-labeled)	In vitro/In vivo preclinical probe construction and target validation in mouse model	Subcutaneous PDAC mouse model (BxPC3: CA19.9+ MiaPaca-2: Ca19.9−)	After 48 h and 120 h (PET/CT)	Tumor uptake/biodistribution in % of injected dosage/g BW (After 48 h; after 120 h)	DFO-5B1 BxPC3 (tumor+): 32; 40 MiaPaCa-2 (control): 8; 7	Proof-of-concept with combined PET/NIRF imaging of CA19.9+ PDAC lesions with 89Zr-ssdual-5B1 to delineate the pancreatic tumor, distant metastases and positive lymph nodes using PET/CT and NIRF imaging. Dual-labeled imaging of CA19.9 with ^89^Zr(ss)dual-5B1 could serve as a guide for the staging, treatment planning, and resection of PDAC	[166]
								Dual-5B1 BxPC3 (tumor+): 36; 45 MiaPaCa-2 (control): 5; 4		
	^89^Zr(ss)FL-5B1									
					Subcutaneous PDAC mouse model (SUIT-2)	After 120 h (single and dual-labeled)	Feasibility of in vivo NIRF-guided resection of tumor, metastases, and suspected lymph nodes	With NIRF-imaging, the tumor, (micro)metastases, and lymph nodes were clearly visible, due to extensive disease no complete resection could be achieved.		
	^89^Zr(ss)dual-5B1									
**Cathep-sin-E**	Ala-Gly-Phe-Ser-Leu-Pro-Ala-Gly-Cys-CONH_2_-Cy5.5	Peptide, linear	NIRF- 700 nm	In vitro/In vivo Preclinical activatable probe construction and target validation in mouse model	Subcutaneous PDAC mouse model (MPanc96-E, CTSE+)	After 24 h, 48 h, 72 h	In vivo Tumor-to-background (SD)	24 h: ±2 48 h: ±2.5 72 h: ±3	Proof-of-concept of an activable NIRF-probe targeting cathepsin-E. Cathepsin-E+ PDAC showed fluorescent signals with subsequent TBR’s (>2) from 24–72 h post-injection.	[60]
							Ex vivo Tumor-to-muscle	16		
	Ala-Gly-Phe-Ser-Leu-Pro-Ala-Gly-Cys-CONH_2_-Cy5.5	Peptide, linear	NIRF- 700 nm	In vitro/Ex vivo Preclinical activatable probe construction and target expression in mouse model	Orthotopic PDAC mouse model (MDA PATC-3, MPanc96-E)	After 48 h	Ex vivo Tumor-to-muscle	5.5	Proof-of-concept of an activable NIRF-probe targeting cathepsin-E. Cathepsin-E+ PDAC. Only Ex vivo quantification has been carried out. Abd-Elgaliel et al. [60] performed additional analysis of the same tracer/probe combination. Adequate signals with TBR’s (>2) from 24–72 h post-injection.	[58]
**CDCP-1**	^89^Zr-DFO-4A06	mAb, hu manized	PET/CT	In vivo Preclinical probe construction and target validation in mouse model	Subcutaneous PDAC mouse model (HPAC, HPAF II, Capan-1, Panc10.05, Panc2.03)	After 72 h	Tumor uptake/biodistribution in % of injected dosage/g BW (SD)	HPAC: 15.21 (±2.2) HPAF II: 7.78 (±4.8) Capan-1: 6.81 (±1.2) Panc10.05: 6.09 (±0.5) Panc2.03: 5.25 (±1.2)	Proof-of-concept, for in vivo PET/CT-imaging of CDCP-1+ PDAC in mice.	[63]
							Biodistribution in % of injected dosage/g BW (range)	Blood: 2–2.5 Muscle: 0.75–1.0		
	^89^Zr-DFO- 10D7	mAb, mouse	PET/CT	In vivo Preclinical probe construction and target validation in mouse model	Subcutaneous/Orthotopic PDAC mouse model (TKCC05)	After 24 h, 48 h, 72 h, 144 h	Tumor uptake/biodistribution in % of injected dosage/g BW (after 24 h)	Subcutaneous: PDAC: 53.0 Liver: 20.0 Spleen: 18.0	Proof-of-concept, for in vivo PET/CT-imaging of CDCP-1+ PDAC in mice.	[146]
							Biodistribution in % of injected dosage/g BW (range)	Blood: 2–2.5 Muscle: 0.75–1.0		
**CEA**	^124^I- anti-CEA scFv-Fc(H310A)	Single-chain variable fragment (scFv-Fc)	PET/CT	In vitro/In vivo Preclinical probe construction and target validation in mouse model	Subcutaneous PDAC mouse model (Capan-1, HPAF-II, and BxPC3)	After 4 h and 20 h	Tumor-to-background ratio (blood pool)	All cell-lines: 4.0 CEA+ Capan-1: 3.7 CEA+ HPAF-II: 3.2 CEA+ BxPC3:5.2	Proof-of-concept of in vivo PET/CT-imaging with a targeted anti-CEA-probe, high specific target binding.	[183]
	Alexa Fluor 488-anti-CEA	mAb, hu manized	Fluorescence imaging- 500 nm	In vivo Preclinical target validation in mouse model	Subcutaneous PDAC mouse model (ASPC-1, BxPC-3, CFPAC, Panc-1, and Capan-1)	After 30 min, 1 h, 2 h, 6 h, 8 h, 24 h, 48 h, 192 h, 360 h	Ability to visualize orthotopic CEA+ pancreatic tumors after 24 h	In vivo fluorescence-imaging re vealed very small pancreatic tu mors which were difficult to visualize using standard brightfield illumination, furthermore extent of tumor invasion could be assessed.	Proof-of-concept of in vivo fluorescence imaging of CEA+ PDAC. Low background due to low CEA expression in normal parenchyma	[218]
					Orthotopic PDAC mouse model (BxPC-3)					
	Alexa Fluor 488-anti-CEA	mAb, hu manized	Fluorescence imaging- 500 nm	In vivo Preclinical CEA+ fluorescence-guided surgery in mouse model	Orthotopic PDAC mouse model (BxPC-3)	After 24 h	Ability to achieve complete resection compared to bright-light-surgery (BLS)	NIRF: 92% (23/25) BLS: 45.5% (10/22)	Proof-of-concept for fluorescence-guided surgery of PDAC, improving complete resection rate and 1-year survival.	[71]
							1-year survival (proportion)	NIRF: 0% (0/22)BLS: 28% (7/25)		
	hM5A-IR800	mAb, hu manized	NIRF- 800 nm	In vitro/In vivo Preclinical probe construction and target validation in mouse model	Orthotopic PDAC mouse model (BxPC-3)	After 6 h, 12 h, 24 h, 48 h, and 72 h	Tumor-to-background ratio (at all time points)	>5.0	Proof-of-concept of in vivo NIRF-imaging of humanized antibody targeting CEA+ PDAC in mice, optimal window after 48 h. Low background fluorescence due to low CEA expression in normal parenchyma. Except for the liver parenchyma, possibly interfering with identification of CEA-positive primary liver or metastatic lesions.	[66]
							Maximum tumor-to-background ratio (at 48 h)	16.6		
**EGFR**	^64^Cu-panitumumab-F(ab’)_2_	Antibody fragment F(ab’)2	PET/CT	In vivo preclinical probe construction and target validation in mouse model	Subcutaneous PDAC mouse model (PANC-1, OCIP23)	After 24 h, 48 h, 72 h	Tumor uptake/biodistribution in % (SD) of injected dosage/g BW (after 24 h-72 h)	PANC-1: Subcutaneous: 11.8 (±0.9) OCIP23: Subcutaneous: 12.0 (±0.9)	Proof-of-concept, high target binding affinity using pani tumumab-F(ab′)_2_ fragments for EGFR+ PDAC allowing tumor visualization during in vivo imaging.	[173]
					PDAC orthotopic tumor bearing mice (OCIP23)			OCIP23 Orthotopic: 6.1 (±1.1) Blood: 2.6 (±0.17) Muscle: 0.3 (±0.02)		
**EGFR/** **VEGF_165_**	Bi50-IRdye800	Diabody	NIRF- 800 nm	In vitro/In vivo preclinical probe construction and target validation in mouse model	Subcutaneous PDAC mouse model (BxPC-3)	After 8 h	Tumor-to-background Ratio (SD)	4.32 (±0.1)	Proof-of-concept, simultane ous excellent target binding capacity to VEGFR and EGFR. Clear delineation of tumor and healthy tissue. Targeting tumor vasculature-rich areas (overexpression of VEGFR), as well as largely bonded the tumor parenchymal cells (EGFR overexpression)	[196]
**FAP**	^18^F-FAPI-74 ^177^Lu-FAPI-46 ^225^Ac-FAPI-46	SMI SMI SMI	PET/CT	In vitro/In vivo preclinical probe construction and target validation in mouse model In vivo preclinical targeted α-emitter therapy efficacy and monitoring validation in mouse model	Subcutaneous PDAC mouse model (PANC-1)	After 1 h,	SUVmean	Tumor: 0.24 (±0.04) Muscle: 0.05 (±0.01) Kidneys: 0.39 (±0.07)	Proof-of-concept, demonstrating the effectiveness of FAP-targeted PET-imaging and therapy in xenograft PDAC mouse model, observing rapid clearance from healthy tissue and high uptake in the tumors 3 h after injection. Tumor-suppressive effects were observed in both PANC-1 xenograft mice treated with [177Lu]FAPI-46 as well as ^225^Ac-FAPI-46, although ^177^Lu-FAPI-46 showed a mild but more prolonged therapeutic effects compared to ^225^Ac-FAPI-46.	[169]
						After 3 h, 24 h	Biodistribution in % of injected dosage/g BW	^177^Lu-FAPI-46 Tumor: 0.36; 0.10 Blood: 0.08; 0.01		
						After 40 d	Therapy effect as relative ratio of tumor size	3 MBq: 0.62 10 MBq: 0.56 30 MBq: 0.27		
						After 3 h, 24 h	Biodistribution in % of injected dosage/g BW	^225^Ac-FAPI-46 Tumor: 0.30; 0.10 Blood: 0.07; 0.01		
						After 30 d	Tumor-suppressive-effect versus control	3 MBq: mild 10 MBq: strong (*p* = 0.05) 30 MBq: strong (*p* = 0.05)		
**Fibronec-tin**	^68^Ga-NOTA-ZD2	Peptide, linear	PET/CT	In vitro/In vivo Preclinical probe construction and target validation in mouse model	Subcutaneous PDAC mouse model (BxPC-3, Capan-1)	After 1 h	Tumor-to-muscle ratio (mouse-1/mouse-2)	BxPC-3: 5.4/5.6 Pacan-1: 10.0/11.0	Proof-of-concept, for in vivo PET/CT-imaging of Fibronectin+ PDAC in mice. ZD2-(68Ga-NOTA) is able to clearly delineate the PDAC with a size of 10 mm or less with minimal background noise in normal tissue, including the liver.	[219]
							BxPC-3: Biodistribution in % of injected dosage/g BW (range)	Tumor: 0.24 Liver: 0.15 Muscle: 0.05		
							Pacan-1: Biodistribution in % of injected dosage/g BW	Tumor: 0.32 Liver: 0.15 Muscle: 0.05		
	^64^Cu-NJB2	Nano-body	PET/CT	In vitro/In vivo Preclinical probe construction and target validation in mouse model	Orthotopic PDAC mouse (BxPC-3, Capan-1)	After 2 h	Tumor-to-muscle ratio	PDAC: 10.0 LN+: 23.0 Liver metastases: 12.0 Muscle: 1.0 Liver: 7.0	Proof-of-concept in a small cohort of mice with orthotopic PDAC, high-affinity target binding of fibronectin using nanobodies, NJB2, allowing for visualization of primary tumor, metastatic lymph nodes and liver metastasis.	[220]
**GRP-78**	^64^Cu-DOTA-MAb159	mAb, mouse	PET/CT	In vitro/In vivo Preclinical probe construction and target validation in mouse model	Subcutaneous PDAC mouse model (BxPC3)	After 1 h, 17 h, 48 h	Tumor uptake/biodistribution in % of injected dosage/g BW	1 h: 4.3 (±1.2) 17 h: 15.4 (±2.6) 48 h: 18.3 (±1.0)	Proof-of-concept, high target binding affinity for GRP78+ PDAC, allowing tumor visual ization with targeted PET/CT-imaging.	[92]
							Tumor-to-muscle ratio (SD)	1 h: 1.40 (±0.30) 17 h: 7.4 (±4.6) 48 h: 11.5 (±7.2)		
**Integrin αvβ3**	^68^Ga-NODAGA-RGD	Peptide, linear	PET/CT	In vivo Preclinical feasibility and target validation in mouse model	Genetically engineered Orthotopic PDAC mouse model (Ptf1a+/Cre;Kras+/LSL-G12D;p53LoxP/LoxP)	After 75 min	Tumor uptake/biodistribution in % of injected dosage/g BW	PDAC: 5.9 Blood: 0.7 Muscle: 0.4	Proof-of-concept, in vivo PET/CT-imaging with high target affinity for αvβ3+ PDAC lesions.	[172]
							Tumor-to-muscle ratio	14.8		
**Integrin αvβ3/αvβ5/αvβ6**	cRGD-ZW800-1	Peptide, cyclic	NIRF- 800 nm	In vivo Preclinical feasibility and target validation in mouse model	Orthotopic PDAC mouse model (BxPC-3)	After 4 h	Tumor-to-background Ratio	PDAC (dose 0.1 nmol): 3.0 PDAC (dose 10 nmol): 3.4 PDAC (dose 30 nmol): 4.0	Proof-of-concept, NIRF-imaging. Clear visualization of PDAC between 2 and 24 h post injection, non-selectively targeting integrins.	[190]
**Integrin αvβ6**	R_0_1-MG-IRDye800	Peptide, cysteine knotted	NIRF- 800 nm	In vivo Preclinical feasibility and target validation in mouse model	Orthotopic PDAC mouse model (BxPC-3, MiaPaCa-2)	After 30 min-24 h	Tumor-to-Background Ratio (SD)	BxPC-3: 2.5 (±0.1) MiaPaCa-2: 1.2 (±0.1) Pdx1-Cretg/+; KRasLSL G12D/+; Ink4a/Arf−/−): 3.6 (±0.94)	Proof-of-concept, NIRF-imaging. High specific affinity for αvβ6, Fluorescent signal and tumor status corresponded well to αvβ6 expression as assessed by IHC. Renal clearance. Suitable for clinical validation in αvβ6+ PDAC.	[192]
					Orthotopic PDAC transgenic mice (Pdx1-Cretg/+; KRasLSL G12D/+; Ink4a/Arf−/−)					
	^68^Ga-DOTA-SFLAP3	Peptide, cyclic	PET/CT	In vitro Preclinical probe construction and target validation in mouse model	Orthotopic PDAC mouse model (Capan-2)	N/A	N/A	N/A	High specific binding affinity to integrin αvβ6 on pancreatic cancer cell lines. No further data available.	[221]* *Only abstract available*
	^68^Ga-cycratide	Peptide, cyclic	PET/CT	Combined pre-clinical probe construction and target validation/Experimental clinical study phase I	2 PDAC patients Orthotopic PDAC mouse model (BxPC-3)	After 30 min	SUVmax	Patient 1: (diagnosis/staging): 4.86, histological confirmation of PDAC. Patient 2: (FU 7 m after surgery and ChemoTx): 1.6, no relapse, inflammatory response.	Proof-of-concept for ^68^Ga-cycratide as effective and selective αvβ6 targeting PET-probe and low-background signal with exclusive renal clearance. Although the clinical part of the study had a small sample size, further evaluation in a clinical setting is needed for the potential of ^68^Ga-cycratide imaging.	[95]
						After 2 h	Tumor uptake/biodistribution in % of injected dosage/g BW (SD)	2.15 (±0.46)		
						After 30 min	Tumor-to-muscle ratio (SD)	4.77 (±1.62)		
**MT1-MMP/MMP-14**	^89^Zr-DFO-LEM2/15 ^68^Ga-DOTA-AF7p	mAb, mouse Peptide, linear	PET/CT	In vitro/In vivo Preclinical probe construction and target validation in mouse model	Subcutaneous PDAC mouse model, (Capan-2)	After 5 d, 7 days	Tumor-to-background (SD) of ^89^Zr-DFO-LEM2/15	5 days: 1.13 (±0.51) 7 days: 1.44 (±0.43)	Proof-of-concept of in vivo PET/CT-imaging of MT1-MMP/MMP-14+ PDAC, with high target specificity for ^89^Zr-DFO-LEM2/15. Low/Moderate specificity for ^68^Ga-DOTA-AF7p. Further evaluation of ^89^Zr-DFO-LEM2/15 is warranted to address its potential in humans.	[222]
					Orthotopic PDX PDAC mouse model	After 90 min	Tumor-to-background of ^68^Ga-DOTA-AF7p	90 min: 0.5		
						After 1 d, 7 days	Tumor-to-blood (SD) of ^89^Zr-DFO-LEM2/15	1 days: 0.56 (±0.10) 7 days: 1.95 (±0.63)		
**Mucin-1**	Anti-MUC1 (CT2)-DyLight550/650	mAb, hamster	Fluorescence imaging- 600 nm	In vitro/In vivo Preclinical probe construction and target validation in mouse model	Subcutaneous/Orthotopic PDAC mouse model (PANC-1, BxPC-3)	After 7–10 days	In vivo Tumor-to-background (Orthotopic tumors)	Panc-1: 6.70 BxPC-3: 2.39	Proof-of-concept of in vivo fluorescence-imaging of MUC-1+ Subcutaneous/Orthotopic tumors. Biodistribution and further evaluation in pre-clinical is warranted before clinical studies could be initiated, furthermore humanized antibodies are preferred over animal antibodies.	[223]
**NTSR1**	^64^Cu-AmBaSar-NT,	Peptide, linear	PET/CT NIRF- 800 nm	In vivo preclinical probe construction and target validation in mouse model	Subcutaneous PDAC mouse model (PANC-1, AsPC-1)	After 30 min, 1 h, 4 h	PET/CT: Tumor uptake/biodistribution in % of injected dosage/g BW (SD)	1 h: 3.76 (±1.45) 4 h: 2.29 (±0.10)	Proof-of-concept, high target binding affinity for NTSR+ PDAC, moderate background in kidney uptake, low background in liver and intestines. Neurotensin peptide sequence could be used for adequate PDAC visualization with PET/CT and NIRF imaging.	[115]
	IRDye800-NT	Peptide, linear			Orthotopic PDAC mouse model (PANC-1, AsPC-1)		NIRF: Tumor-to-background Ratio (SD)	30 min: 8.09 (±0.38) 1 h: 6.67 (±0.43)		
	^68^Ga-DOTA-NT-20.3	Peptide, linear	PET/CT	In vivo preclinical probe construction and target validation in mouse model	Subcutaneous PDAC mouse model (AsPC-1)	After 45 min	SUV_max_ (SD)	Subcutaneous: PDAC: 1 (±0.2) Background: 0.3 (±0.1)	Proof-of-concept, high target binding affinity for NTSR+ PDAC, adequate tumor-to-background ratio. Moderate background in kidney uptake, low background in liver and intestines. DOTA-NT-20.3 distinguishes PDAC from pancreatitis in orthotopic mouse model. NT-20.3 receptor targeting peptide sequence could be used for adequate PDAC visualization with PET/CT imaging.	[116]
						After 45 min	Tumor-to-no-tumor ratio (SD)	Subcutaneous: 3.5 (±0.8)		
					Orthotopic PDAC mouse model (AsPC-1)	After 1 h	Tumor-to-blood ratio	Subcutaneous: 6.0		
						After 1 h	Tumor uptake ratio (SD)	Orthoptic: 4.6 (±1.5)		
**TF**	^64^Cu-NOTA-FVIIai	SMI	PET/CT	In vivo Preclinical feasibility and target validation in mouse model	Subcutaneous PDAC mouse model (PANC-1, AsPC-1, BxPC-3)	After 36 h	Maximum tumor uptake/biodistribution in % of injected dosage/g BW	PDAC: 3.7 Pancreas: 0.3 Liver: 8.0 Blood: 0.2 Muscle: 0.1	Proof-of-concept, high accumulation in PDAC, suitable for PET/CT-imaging of TF+ PDAC. High accumulation in Liver, possibly interfering with imaging of the PDAC lesion due to high background. Delayed imaging of 64Cu-NOTA-FVIIai improved the tumor–to–background ratio, and subcutaneous tumors were clearly visible 15 h after injection.	[131]
						After 15 h, 36 h	Tumor-to-muscle ratio	After 15 h: 20		
							Tumor-to-pancreas ratio	After 36 h: 36 After 15 h: 10 After 36 h: 13		
							Maximum tumor uptake/biodistribution in % (SD) of injected dosage/g BW	PANC-1 (low TF+): 2.2 (±0.1) AsPC-1 (intermed. TF+): 4.1 (±0.1) BxPC-3 (high TF+): 7.5 (±0.5)		
**TF/** **Endoglin (CD105)**	^64^Cu-NOTA-ALT-836/TRC105	Dual- targeted antibody fragment	PET/CT	In vitro/In vivo Preclinical probe construction and target validation in mouse model	Subcutaneous PDAC mouse model (BXPC-3, TF/CD105+/+)	After 30 h	Tumor uptake/biodistribution in % of injected dosage/g BW (SD)	Subcutaneous: PDAC: 17.1 (±4.9) Pancreas: <1 Liver: 8.5	Proof-of-concept, high target binding affinity for dual-TF+/Endoglin+ PDAC, allowing tumor visualization with targeted PET/CT-imaging. Renally cleared.	[197]
					Orthotopic PDAC mouse model (BXPC-3, TF/CD105+/+)	After 30 h	Tumor-to-muscle ratio (SD)	Orthotopic: 72.3 (±46.7)		
**Trans-ferrin receptor-1 (TfR1)**	^89^Zr-TSP-A01	mAb, hu manized	PET/CT	In vitro/In vivo Preclinical probe construction and target validation in mouse model	Subcutaneous PDAC mouse model (A4, MiaPaCa-2)	After 24 h; 6 days	Tumor-to-muscle ratio (Mouse 1/Mouse 2)	PDAC MiaPaCa-2: Day 1: 4.6/4.7 Day 6: 10.7/8.6	Moderate/High target affinity, promising PET tracer to detect TfR1+ PDAC, although only tumors of MiaPaCa-2 cell line were clearly visualized. Moderate uptake in healthy liver parenchyma.	[224]
								PDAC A4: Day 1: 2.2/2.4 Day 6: 2.1/2.6		
						After 6 days	Maximum tumor uptake/biodistribution in % of injected dosage/g BW	PDAC MiaPaCa-2: 12.0 PDAC A4: 4.0 Blood: 4.0 Muscle: <1.0		
**uPA/** **uPAR system**	^89^Zr-Df-ATN-291	mAb, hu manized	PET/CT	In vitro/In vivo preclinical probe construction and target validation in mouse model	Subcutaneous PDAC mouse model (BxPC-3)	After 2, 24, 72 and 120 h	Tumor-to-muscle Ratio (after 24, 72 h)	PDAC: 7.4–21.3	Proof-of-concept, high target affinity for uPAR+ PDAC, useful imaging tool for cancer (metastasis) detection and evaluation of a given uPA/uPAR-targeted treatment.	[174]
							Tumor uptake/biodistribution in % (SD) of injected dosage/g BW (after 24 h-72 h)	PDAC: 9.4 (±0.6)–18.9 (±1.9)		
	Glu-Glu-AE105-ICG	Peptide, linear	NIRF- 800 nm	In vivo preclinical target validation and NIRF-guided surgery in mouse model	Orthotopic PDAC mouse model (BxPC-3)	After 15 h	Tumor-to-background Ratio (95% CI)	PDAC: 3.5 (3.3–3.7) Metastases: 3.4 (3.1–4.0)	Clear localization of primary PDAC and metastases with NIRF imaging Glu-Glu-AE105-ICG. Identification of additional fluorescent lesions, resulting in resection.	[193]
							Identification and removal of additional metastases only on NIRF compared (%)	Mice: 4 out of 8 (50%) Metastases: 6/35 (14%)		

An overview is given of preclinically evaluated PDAC targeted PET/(NIR)Fluorescence imaging agents, categorized on alphabetical order. Main study design features, results, and highlights are shown. Abbreviations: CA19.9 = Carbohydrate antigen 19.9; CDCP1 = CUB domain-containing protein-1; CEA = Carcinoembryonic antigen; EGFR = Epidermoid growth factor receptor; EpCAM = Epithelial cell adhesion molecule; FAPα = Fibroblast-activating protein-α; FGS = Fluorescence-guided surgery; GRP78 = Glucose-regulating protein-78; IPMN = Intraductal papillary mucinous neoplasm; IQR = Inter-quartile range; LN = Lymph node; mAb = Monoclonal antibody; MMP = Matrix metalloproteinase; NET = Neuro endocrine tumor; NIRF = Near-infrared Fluorescence; NPV = Negative predictive value; NTSR-1 = Neurotensin receptor-1; PDAC = Pancreatic ductal adenocarcinoma; PPV = Positive predictive value; PSMA = Prostate membrane antigen; SD = Standard deviation; SMI = Small Molecule Inhibitor; SUV = Standardized uptake value; TBR = Tumor-to-background ratio; TfR1 = Transferrin receptor; TF = Tissue Factor; uPa = Urokinase-type plasminogen activator; uPAR = Urokinase-type plasminogen activator Receptor; VEGFR(2) = Vascular endothelial growth factor receptor; VEGF-α = Vascular endothelial growth factor α.
